# Colorimetric Signal Readout for the Detection of Volatile Organic Compounds Using a Printable Glass-Based Dielectric Barrier Discharge-Type Helium Plasma Detector

**DOI:** 10.1021/acsmeasuresciau.3c00012

**Published:** 2023-05-30

**Authors:** Jingqin Mao, Longze Liu, Yahya Atwa, Junming Hou, Zhenxun Wu, Hamza Shakeel

**Affiliations:** † School of Electronics, Electrical Engineering and Computer Science, Queen’s University Belfast, Belfast BT7 1NN, U.K.; ‡ State Key Laboratory of Millimeter Waves, School of Information Science and Engineering, Southeast University, Nanjing 210096, China; § Queen’s Management School, Queen’s University Belfast, Belfast BT7 1NN, U.K.

**Keywords:** dielectric barrier discharge plasma, photoionization detector, volatile organic compound, gas chromatography, image process, image light intensity change

## Abstract

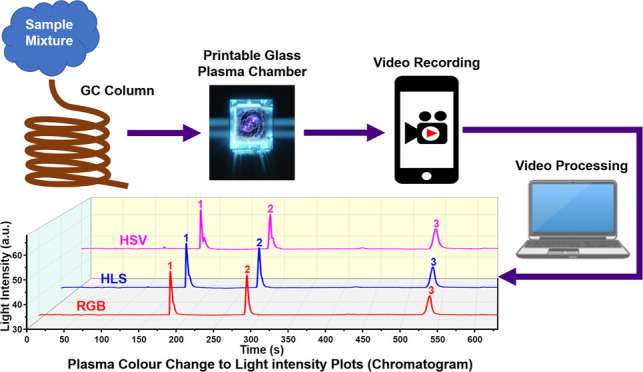

In this paper, we report on a printable glass-based manufacturing method and a new proof-of-concept colorimetric signal readout scheme for a dielectric barrier discharge (DBD)-type helium plasma photoionization detector. The sensor consists of a millimeter-sized glass chamber manufactured using a printable glass suspension. Plasma inside the chip is generated using a custom-built power supply (900 V and 83.6 kHz), and the detector uses ∼5 W of power. Our new detection scheme is based on detecting the change in the color of plasma after the introduction of target gases. The change in color is first captured by a smartphone camera as a video output. The recorded video is then processed and converted to an image light intensity vs retention time plot (gas chromatogram) using three standard color space models (red, green, blue (RGB), hue, saturation, lightness (HSL), and hue, saturation, value (HSV)) with RGB performing the best among the three models. We successfully detected three different categories of volatile organic compounds using our new detection scheme and a 30-m-long gas chromatography column: (1) straight-chain alkanes (*n*-pentane, *n*-hexane, *n*-heptane, *n*-octane, and *n*-nonane), (2) aromatics (benzene, toluene, and ethylbenzene), and (3) polar compounds (acetone, ethanol, and dichloromethane). The best limit of detection of 10 ng was achieved for benzene at room temperature. Additionally, the device showed excellent performance for different types of sample mixtures consisting of three and five compounds. Our new detector readout method combined with our ability to print complex glass structures provides a new research avenue to analyze complex gas mixtures and their components.

## 1 Introduction

Gas chromatography (GC) is an analytical tool widely used for several applications including medicine, biomarker identification for health monitoring, petrochemical industry, environmental monitoring, food processing, and biochemistry.^1−5^ Photoionization detectors (PIDs) demonstrate excellent performance for the detection of volatile organic compounds (VOCs) and are widely used in both conventional GC and micro gas chromatography (μGC) systems.^6−11^ Several PID designs have been reported in the literature, and the current research interests are focused mainly on the miniaturization of PIDs.^12^ For a typical PID configuration, the core components include an ionization source, ionization chamber, and output signal readout mechanism. The ionization source of PIDs is typically a lamp filled with a rare gas (e.g., argon, krypton, and xenon) to generate photons with high energy (8.3–11.8 eV).^9,12,13^ These light sources are then used to ionize chemicals with ionization potential (IP) below 11.8 eV. Moreover, lamp-based PIDs are either used independently or can be coupled with GC systems for the detection of complex sample mixtures. For compounds with higher IP, such as methane (IP: 12.98 eV), propionitrile (IP: 11.84 eV), and chlorine trifluoride (IP: 12.65 eV), the detection capability of these PIDs is limited.^9^ Comparatively when helium plasma is used as an ionization source, PID can provide photons with higher energy (13.5–17.5 eV) due to the Hopfield emission.^9,14−20^ Compared with lamp-based PIDs, the high photon energy generated by helium discharge-based PIDs (HD-PIDs) makes them excellent candidates for hard-to-ionize gases. HD-PID is usually used in atmospheric pressure operating conditions.^9,18,21^ The plasma excitation methods in HD-PID are based on direct current (DC) discharge,^10,22−24^ pulsed discharge,^25−28^ or dielectric barrier discharge (DBD).^9,20,29,30^ Among these methods, the DBD-based helium plasma has the advantages of providing uniform discharge^29,31^ and long electrode lifetime.^32^ DBD was invented by Siemens in 1857 and the plasma generated by DBD is nonthermal plasma.^33,34^ The structure of DBD is relatively simple, a typical DBD configuration includes a dielectric material, discharge chamber, and metal electrodes and is driven by an alternating current power source at a high frequency (kHz to several MHz) and a high AC voltage amplitude (1–100 kV).^9,35−37^

Several HD-PID designs have recently been reported for both conventional GC and μGC systems, exhibiting excellent detection sensitivities. In 2016, Zhu et al. reported a chip-based micro-HD-PID (μHDBD-PID) with an excellent detection limit, a large linear dynamic range, and low manufacturing cost.^9^ Later in 2018, the group further extended the application of μHDBD-PID to 2D-GC, achieving accurate and repeatable results for formaldehyde detection.^38^ In 2021, Li et al. proposed a reproducible and robust method for batch fabrication of μHD-PID, requiring low power and helium consumption, with a limit of detection <10 pg.^20^ Moreover, previous studies on μHD-PIDs and other μGC detectors, such as thermal conductivity,^39,40^ capacitive,^41,42^ and chemiresistive,^43−45^ are based on the acquisition and processing of electrical readout signals as an equivalent output for chemical detection. Typically, this output signal is a current signal captured via a separate set of electrodes and a DC power supply.

Traditional μGC detectors use a gas detection scheme that is ″blind″ to the observer, which means that they cannot identify the specific chemicals present in the analyzed sample. However, some nontraditional gas detector designs have been reported that may offer improved chemical identification capabilities.^46−50^ For instance, Bulbul et al. reported a nontraditional gas detector for a GC system.^46^ Their sensor utilized the unique relationship between liquid bubble diameter, gas type, and mixing ratio as a sensing mechanism. The gas detection process was carried out by visually observing the bubble generation process using an optical camera. In 2008, Shopova et al. reported on an on-column μGC detector that utilized capillary-based optical ring resonators to detect gases by detecting the refractive index change caused by the interaction between the gas sample and the stationary phase.^47^ In 2010, Liu et al. developed a μGC detector that detected different gases by measuring the change of refractive index of the polymer coated on the Fabry–Pérot sensor. The detector was used for multipoint on-column detection.^48^ In 2018, Du et al. described a surface plasmon resonance imaging technology for the μGC system.^49^ In 2020, Qin et al. developed a paper sensor coated with a polymer that could detect light hydrocarbon gases using a smartphone camera, showing the potential of smartphones for gas detection.^50^ Furthermore, nontraditional sensing mechanisms based on biomaterials have also been reported. In 2007, Potyrailo et al. measured the reflectance spectra of the Morpho sulkowskyi butterfly and found that its iridescent scales could produce different optical responses to individual vapors such as water, methanol, ethanol, and isomers of dichloroethylene.^51^ This work inspired several follow-up studies.^52−61^ These nontraditional detection schemes demonstrate the potential to identify different gases in a complex sample mixture. However, these designs usually require a relatively large amount of analyte to generate the output signal with a sufficient signal-to-noise ratio, resulting in an inferior limit of detection (LoD) compared to detectors based on traditional electrical readout methods. Additionally, the complex nature of their prototype designs has prevented the commercialization of these nontraditional gas detectors.

**Figure 1:**
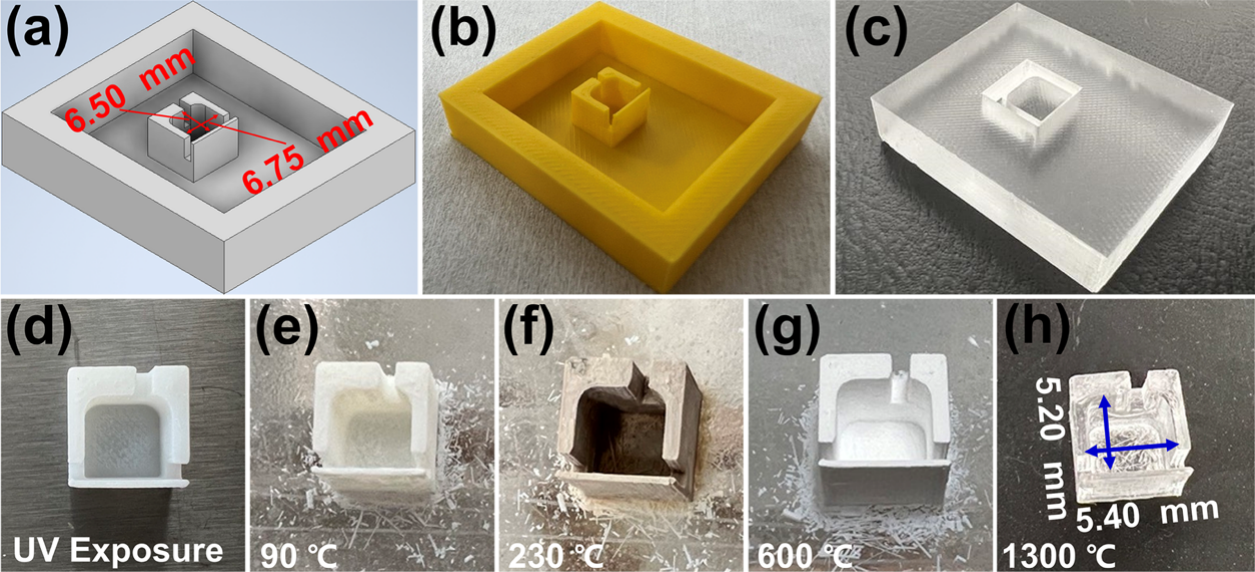
Fabrication flowchart of μHDBD-PID plasma chamber. (a) Three-dimensional (3D) design used for printing plastic mold. (b) 3D-printed positive tone template. (c) Negative-tone PDMS template used for casting. (d) UV-cured Glassomer chamber. (e) Device after first thermal debinding step performed at 90 °C. (f) Device after second thermal debinding performed at 230 °C. (g) Chamber after final debinding step carried out at 600 °C. (h) Final fused silica device after sintering step performed at 1300 °C for 2 h.

To date, there have been no reports on the utilization of colorimetric readout for gas detection for μHD-PIDs. In this work, we present a μHDBD-PID that utilizes the change in image light intensity of ignited plasma upon sample introduction as a signal output method. This change in plasma color is first captured via smartphone as a video. The recorded video is then used to correlate frame intensity changes to chromatography peak width and elution times using three standard video processing techniques. The plasma chamber of our device is fabricated using a commercially available printable glass suspension and enables the manufacturing of complex geometry (Figure 1). The dimensions of the plasma chamber are 5.4 mm × 5.2 mm × 5.0 mm (length × width × height) and is driven by an AC power supply source with an amplitude of ∼900 V and a frequency of 83.6 kHz.^20^ The viewing glass window attached separately to the printable plasma generation glass chamber is highly transparent with a thickness of 0.17 mm and ensures that the video signal can be clearly captured by a smartphone camera. All target gases have strong emission lines within the visible spectrum and generate luminescence when ionized by plasma. The device successfully demonstrated the detection of alkanes, aromatics, and polar organic compounds. Moreover, our new detection scheme can also detect multicomponent VOC mixtures when coupled with a GC column.

The videos recorded by smartphone camera are processed frame by frame by Python code using image processing techniques. We calculate and compare the average brightness of each frame and generate an equivalent chromatographic output peak using three different color space models. These models include red, green, blue (RGB); hue, saturation, lightness (HSL); and hue, saturation, value (HSV). An integrated computer software with live frame to image light intensity conversion, video recording, and video processing capabilities is currently under development to realize real-time measurements. To the best of our knowledge, we are the first group to use image processing techniques to generate an output signal from a μHDBD-PID. Moreover, the use of the colorimetric method as a readout for plasma detection provides a new research direction compared to the traditional and commonly used electrical readout methods.

## 2 Experimental Section

### 2.1 Materials

We purchased *n*-pentane (≥99%), *n*-hexane (≥99%), *n*-heptane (≥99%), *n*-octane (≥99%), *n*-nonane (≥99%), benzene (99.8%), toluene (99.8%), ethylbenzene (99.8%), ethanol (absolute), acetone (≥99.8%), and dichloromethane (≥99.8%) from Sigma-Aldrich (U.K.) and used in experiments without any subsequent purification. Air samples were collected from the laboratory using a gas-tight syringe. High-purity compressed helium (99.999%, Grade Zero, N5.0) is supplied by BOC Gases (U.K.). A silicone elastomer kit (SYLGARD 184) including poly(dimethylsiloxane) (PDMS) and matched hardener was bought from Dow Silicones, U.K. A printable glass casting solution (UV-L50) with an appropriate hardener was ordered from Glassomer GmbH (Germany). A silver conductive epoxy adhesive (Loctite Hysol 9492 and 8331 adhesive) and heatsink compound (metal oxide, 0.65 W/m·K) were purchased from RS Components Ltd (U.K.). An ultraviolet (UV) curable glue (Norland Optical Adhesive 81) was purchased from Edmund Optics (U.K.). We purchased ultrathin transparent glass plates with a diameter of 30 mm and a thickness of 0.17 mm from Thermo Scientific (U.K.). A clear acrylic sheet with a thickness of 3 mm was purchased from Rapid Electronics (U.K.). The flexible fused silica capillary tubing, which has an inner diameter of 150 μm and is used to provide chip-to-GC interface, was purchased from Molex, U.K. A 30-m-long GC Column (Agilent J&W HP-5, 0.32 mm internal diameter and 0.25 μm thick coating layer) was purchased from the Agilent Technologies company and is used for all of the experiments.

### 2.2 Characterization

All infrared thermal images were captured by an FLIR SC640 thermal camera. A Keyence (VHX-7000) 4K high-accuracy digital microscope (U.K.) was used to take microscopic images to characterize the surface profile of the glass viewing window. An Agilent GC system (7820A) with an automatic pressure controller and split/split-less sample injection function was used for all experiments.

### 2.3 Device Fabrication

Figure 1 shows the design and fabrication of our plasma generation chamber, which was created using a printable glass suspension and 3D printing. The geometry of the plasma chamber is generated using a 3D CAD software (Figure 1a), and to account for device shrinkage due to multiple thermal processing steps, we conducted three different experiments and found that the final device shrinks 20% isometrically compared with the initial design. Therefore, the initial chamber was designed as 6.75 mm × 6.5 mm × 6.25 mm (length × width × height) to achieve the target size (5.4 mm × 5.2 mm × 5.0 mm). Figures S1 and S2 provide a detailed overview of the design process and dimensions of the positive tone mold used for 3D printing. The dimensions of the plasma chamber in our device were determined based on multiple factors, particularly the height. We used a length and width of 5.4 mm × 5.2 mm, which are similar to those used by Li (5.0 mm × 4.5 mm)^20^ and were slightly modified to accommodate our fabrication limitations. The choice of a height of 5 mm was motivated by several factors. First, our tests required a relatively large observation window to measure the changes in helium plasma light intensity. Additionally, since our devices were manually fabricated, it was challenging to create small-sized devices. Moreover, when the height of the chamber is greater than 3 mm, it is easier to cut and assemble the observation window. Li et al. developed an HD-PID with a height of 0.75 mm.^20^ However, a height of 0.75 mm for the glass observation window is difficult to cut manually and too narrow for plasma observation. To determine the optimal height for our device, we used COMSOL Multiphysics to simulate the maximum electron density for different electrode spacings ranging from 0.2 to 10 mm. The simulation results showed that the maximum electron density occurs at a spacing of approx. 1.3 mm, indicating that a device height greater than 0.75 mm is preferable. Moreover, there was no significant change in maximum electron density when the spacing was increased from 2 to 5 mm. However, since our existing process does not easily allow us to produce devices with heights less than 5 mm, we decided to use a height of 5 mm for our devices.

The fabrication process of the plasma generation chamber started with 3D printing (Figure 1b) the design using a commercial printer (UltiMaker S5, the Netherlands) and PolySmooth as the print material. We then poured a mixture of PDMS and hardener (mass ratio of 15:1) into the 3D-printed template to create a negative-tone polymer mold. After removing bubbles from the mixture using a vacuum desiccator (Bel-Art SP Scienceware Lab Companion), it was left to solidify for 72 h at room temperature (Figure 1c). In the next step, we slowly poured the liquid Glassomer suspension into the negative-tone PDMS mold. An acrylic sheet was placed on the top of the PDMS mold with Glassomer and irradiated under a UV lamp (DYMAX BlueWave 200 system, Dymax Corporation) for 300 s with an intensity of 7.5 mW/cm^2^. It is important to note that directly using a 3D-printed negative-tone plastic mold for casting results in the stiction of printable glass suspension. The final UV-cured Glassomer chamber is shown in Figure 1d.

The Glassomer chamber underwent thermal debinding and sintering processes. The first debinding process was completed using an oven (Heraeus D-6450, Germany) coupled with a PID power controller (Eurotherm, U.K.) at 90 °C for 6 h (Figure 1e). The second and third debinding steps were performed at 230 °C and 600 °C (both for 6 h), respectively, using a tube furnace (Tempress Lindberg, Japan). The images of glass chamber after two debinding steps, used to remove the binding polymer, are shown in Figure 1f,g, respectively. It is worth mentioning here that the device at this stage is extremely fragile and requires extreme care during handling. Finally, the sintering process was carried out by a high-temperature furnace (Nabertherm, Germany) at 1300 °C for 2 h. The final fabricated fused silica plasma chamber is shown in Figure 1h with the device shrinking by 20% compared to the initial design.

### 2.4 Device Assembly

**Figure 2:**
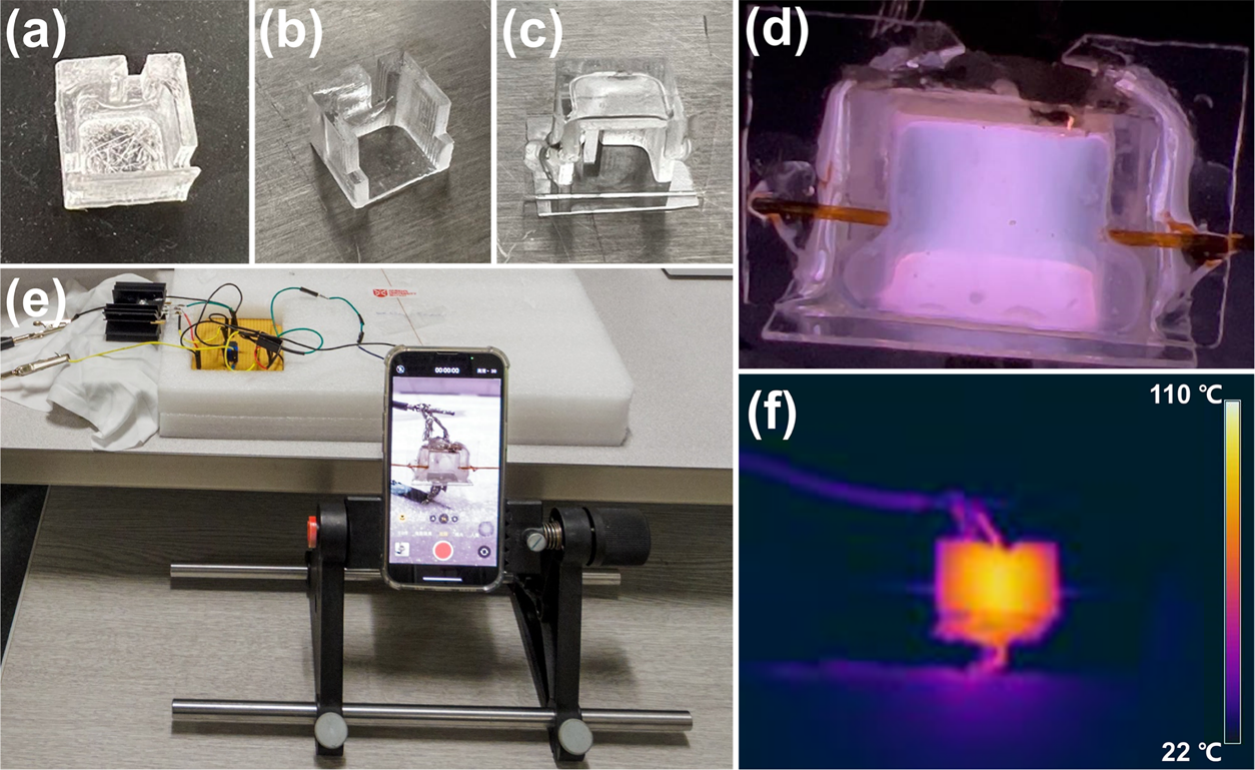
(a) Image of glass chamber taken directly after sintering process. (b) Chamber after cleaning with deionized water and removal of residue using a small brush. (c) Fully assembled chamber after attachment of thin glass slides (bottom slide is used for metal electrode and front slide as a viewing window). (d) Image of a working device demonstrating a uniform plasma within device chamber. (e) Test setup of μHDPID with AC power supply and smartphone used to capture video. (f) Thermal image of device during operation shows a maximum temperature of ∼103 °C.

As shown in Figure 2a, there was a significant residue inside the device after sintering step. Therefore, we first cleaned the device with deionized water and a soft brush. The cleaned glass chamber surface is smooth and transparent (Figure 2b). Afterward, the top dielectric plate and viewing window are cut from thin glass plates and attached to the cleaned chamber (Figure 2c) using a UV-curable glue (Norland 81) under a UV lamp with an energy intensity of 7.5 mW/cm^2^ for 300 s. Next, conductive silver epoxy (8331) and adhesive (Loctite Hysol 9492) are mixed in a mass ratio of 5:1 and stirred evenly. The surfaces of both top and bottom dielectric glass plates of the assembled device were coated with the silver epoxy mixture to provide electrical connections. Afterward, the plasma chamber with metal electrodes and attached metal wires was carefully transferred to an oven and heated at 65 °C for 20 min. Finally, the detector was connected to a conventional GC column and two short uncoated capillary tubes are also attached to act as gas outlets using UV-curable glue (Figure S3). The final assembled μHDBD-PID was then connected to a custom-built AC power supply.

### 2.5 Design of Power Supply

The power supply design used for our device is based on the work published by Li et al. (Figure S4a).^20^ We used the same astable multivibrator (NE555) IC and transformer (ZS1052(H)) as Li et al. but replaced the n-channel silicon MOSFET (IRF740SPBF) with a silicon carbide (SiC)-based MOSFET (SCT30N120, STMicroelectronics) due to overheating issues with the silicon MOSFET. The high working temperature of silicon MOSFET resulted in the melting of solder, so we mounted a SiC power MOSFET on a heatsink (SK129–38,1-STS-220, Fischer Elektronik) as shown in Figure S4b. We used a benchtop DC power supply (PL320 Thurlby Thandar Instruments) to supply 5 W of power (10 VDC and 0.5 A). Thermal images of the custom-built A.C. power supply unit during operation are shown in Figure S4c.

After using the SiC MOSFET mounted on a heatsink, the maximum operating temperature of the MOSFET was 39.6 °C, while the NE555 IC heated up to ∼54 °C, and the transformer reached a temperature of 143 °C. This clearly shows that a lot of electrical energy is being dissipated by the transformer.^20^Figure 2d,f shows the generated helium plasma and its thermal image with a working temperature of about 103 °C. Our device can easily operate for 10 h of continuous operation with an input power of 5 W. However, increasing the input voltage and current can further improve the plasma density, but it can also result in permanent damage to the transformer. Therefore, we are currently working on an improved power supply design to reduce power consumption and improve the lifetime of the device.

### 2.6 Experimental Setup

Figure 2e shows our experimental test setup, which includes an AC power supply, 3D-printed plasma generation chamber, and a smartphone used for video recording. To characterize μHDBD-PID, it was connected to a GC system (Agilent 7820A) through a 30-m-long GC column. All VOCs were manually injected in liquid form through the GC injection port under split mode, and high-purity helium was used as a carrier gas. The inlet temperature of the GC injection port was set to 270 °C, and the pressure was set to 20 Psi. An iPhone 13 Pro smartphone with 512 GB memory was used to record each experiment from injection to final elution.

Each chemical was first independently injected to measure the elution time through the GC column. The iPhone was set to HD with a 30-frames-per-second shooting mode, and the distance between the iPhone lens and the plasma chamber was fixed at ∼1 cm, as a shorter distance can result in electromagnetic interference originating from high-voltage AC source terminals. The videos recorded by the iPhone were later processed by Spyder software (Anaconda) based on customized Python code.

### 2.7 Python Program Operation

The videos recorded by smartphone were first encoded by MPEG-4 into MOV format.^62^Figure S5 gives a detailed flow of video processing based on Python. Briefly, the VideoCapture class in the OpenCV module is used to read the stored video in the specified folder, and the cv2.VideoCapture.IsOpened() function is used to check whether the video is opened or not. Next, the cv2.VideoCapture.Read() function checks the video frame of the opened video. The returned frame is a third-order ndarray that contains the frame length, width information, and RGB information of each pixel.^63^ Afterward, NumPy and Python Colorsys modules are used to compute the values of R, G, B, Y, L, and V of each frame, and results are appended to their respective lists.^64^ For image light intensity, the value of Y of each image frame can be calculated using the following equation:^65^
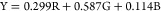
1In HSL and HSV color spaces, L and V represent the image light intensity, and both range from 0 to 1. They can be calculated based on RGB converted values using the Python colorsys module. Since the value of Y is between 0 and 255, so we multiply L and V with 255 in the program to compare these values with Y. The relationship between Y, L, and V and the video frame number is plotted using Python Matplotlib, and all of the data is saved in a comma separated values (csv) file (Figure S5). Our program can process multiple videos in one folder, and the detailed Python code is attached in the Supporting Information (Python Code for Video Processing). Furthermore, considering the time-consuming transfer process from video recording to video processing, we are currently developing computer-based software for monitoring and recording plasma light intensity change using an external camera. Our first-generation app can conduct image processing and video recording simultaneously.

### 2.8 MATLAB Program

To obtain images with specific frame numbers (time points), MATLAB software was separately used to process the videos. The videos captured by the iPhone were transferred to the computer via a USB-C cable. Then, the MATLAB program utilized the VideoReader object to read the videos and decompose them into 30 frames per second. All of the plasma images presented in this paper were obtained using this method, and the MATLAB code is also available in the Supporting Information (MATLAB Code).

## 3 Results and Discussion

### 3.1 Helium Flow and Plasma Simulation

**Figure 3:**
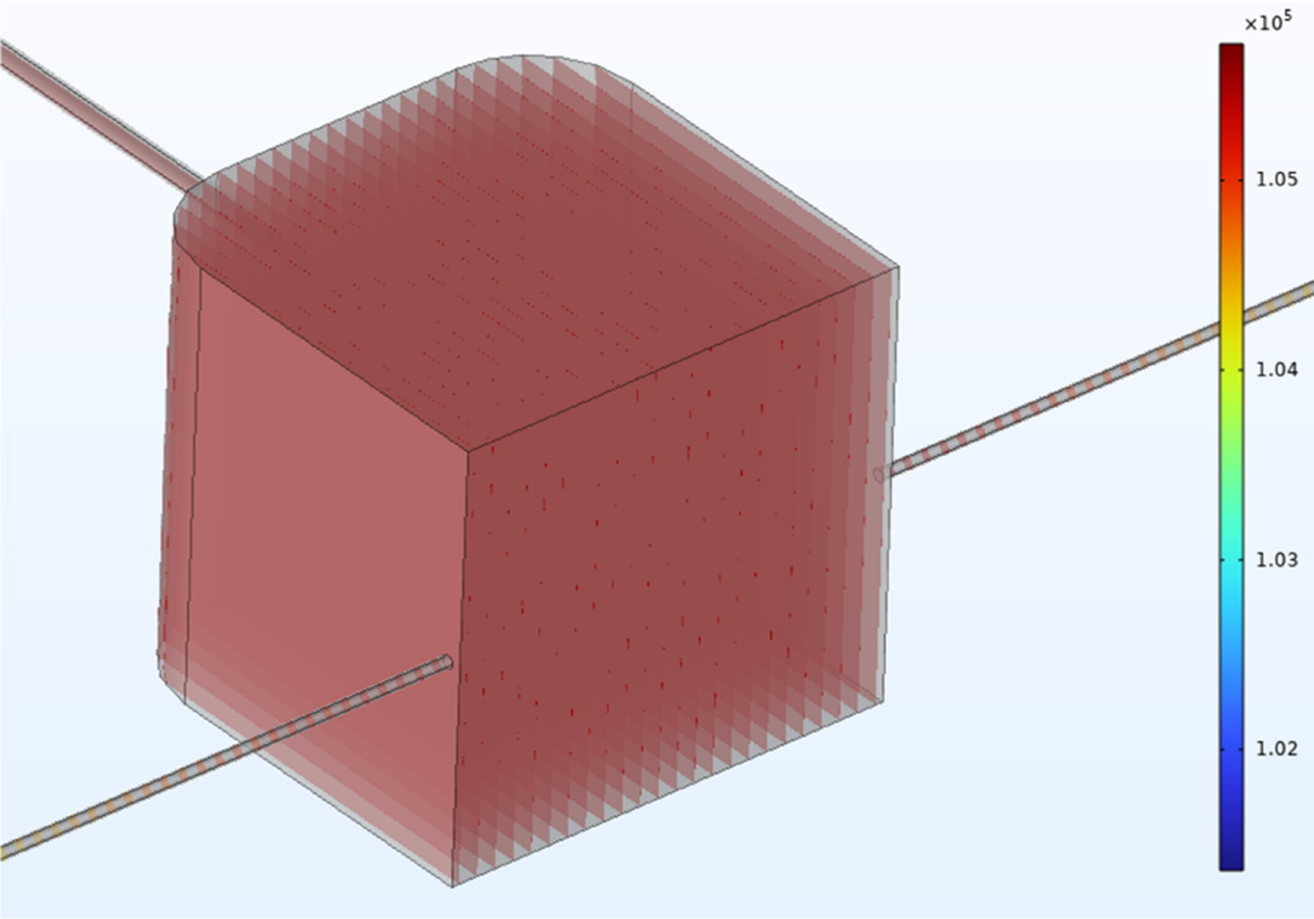
Simulation of 3D pressure profile of helium flow inside the plasma chamber (unit: Pa).

The helium flow and electron density inside the plasma chamber were simulated using the plasma module within the COMSOL Multiphysics environment. The actual pressure of helium inside the chamber is an essential parameter for plasma simulation. Therefore, we first built a 3D flow model based on the actual device dimensions (Figure S6). The simulation results show that the helium pressure inside the chamber is ∼104,700 Pa (Figure 3), and the helium flow velocity can reach up to 0.45 m/s (Figure S7a). Figure S7b shows the helium flow inside the plasma chamber (colored by velocity field). Based on the simulation results of the 3D helium flow simulation, we built one-dimensional (1D) and two-dimensional (2D) DBD models. Figure S8 shows the selection of 1D and 2D simulation areas (Figure S8a) and the construction of the models (Figure S8b,c). Since the helium pressure in the chamber is close to atmospheric pressure, the initial electron density was set to 1 × 10^13^ m^–3^, and the mole fraction of helium metastable (Hes) was set to 1 × 10^–8^.^66^ The chemical reactions involved in plasma simulation are listed in Table 1.^67^

**Table 1:** Reactions Considered in Helium Plasma Simulations

number	reaction	rate coefficient	Δε (eV)
1	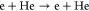 1	BOLSIG+	
2	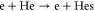 2	BOLSIG+	19.8
3	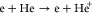 3	BOLSIG+	24.6

**Figure 4:**
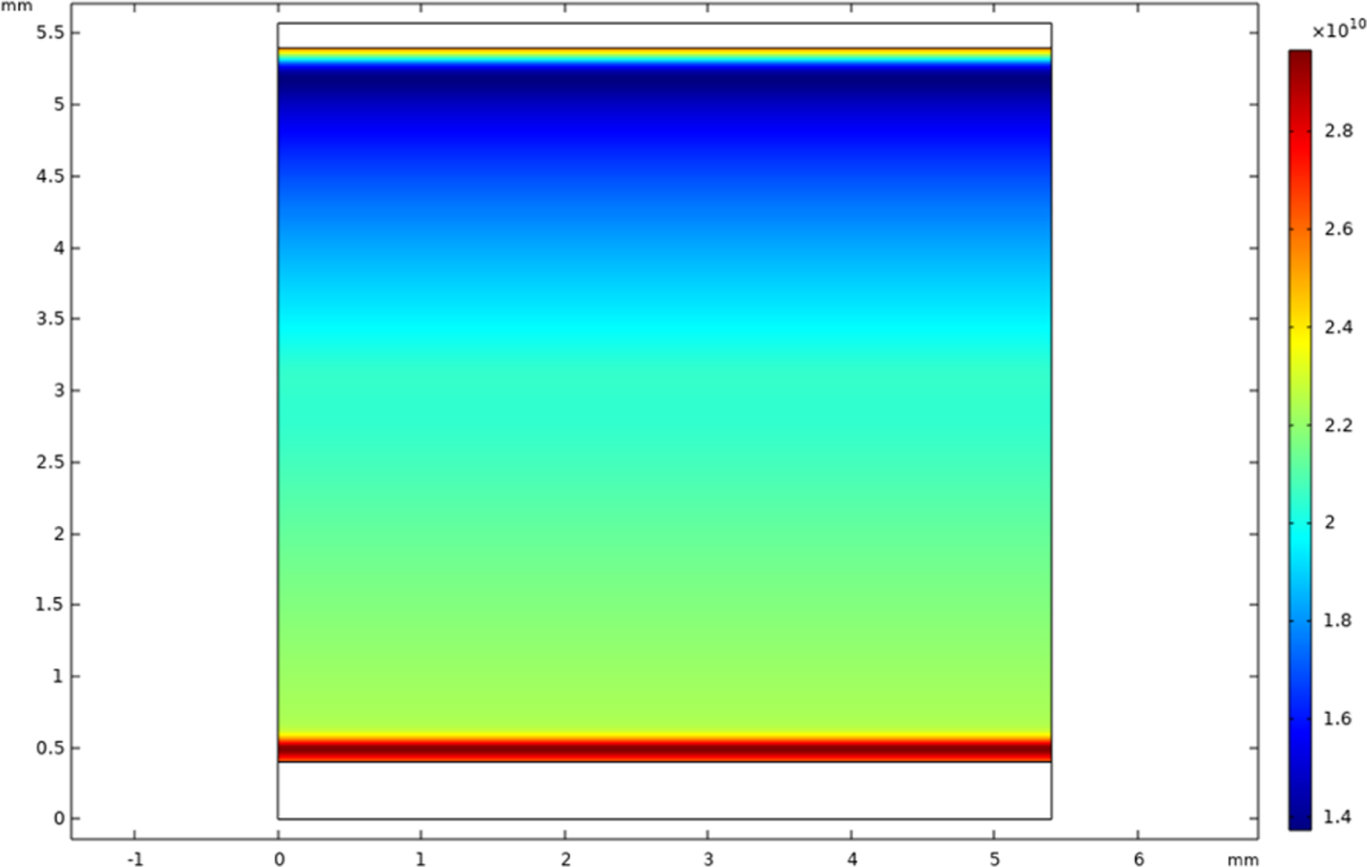
2D simulation results show helium plasma electron density distribution at the 10th discharge cycle and confirm plasma stability at 900 V AC signal with a frequency of 83.6 kHz.

Figure 4 shows a 2D plot of helium plasma electron density distribution at the 10th discharge cycle, and as we can see, the high-density electron area is close to the top and bottom dielectric plate surfaces. During the 10th discharge cycle, the maximum value of electron density obtained by both 1D (Figure S9a) and 2D plasma models is ∼3 × 10^10^ m^–3^. Both 1D and 2D maximum electron densities are lower than the reported atmospheric DBD helium plasma electron density magnitude (range from 10^14^ to 10^20^ m^–3^).^66,68−71^ One main reason is that the power supply voltage amplitude of our device is much lower than the reported values (0.9 kV vs more than 10 kV). Another reason is that our device has a larger discharge gap (5 mm). Low-voltage amplitude and large discharge spacing create a relatively lower electric field strength in the plasma chamber and result in a low density of electrons. When we set the voltage amplitude to 9 kV in our model, the simulated electron density reaches 1.1 × 10^18^ m^–3^ (Figure S9b) as reported in the literature.^66,71^ This clearly shows that a higher voltage can generate a higher density of electrons within our device and validates our simulation model.

Moreover, we have also confirmed with both simulations and experiments that a voltage amplitude of less than 700 V does not generate a stable plasma within our device. It is worth noting that a higher voltage will result in higher power consumption and a larger AC power supply unit. We are currently in the process of further optimizing our design based on simulations for the next generation of devices while keeping in mind both fabrication and signal readout constraints of our new detection scheme.

### 3.2 Change in Plasma Light Intensity Measurements

**Figure 5:**
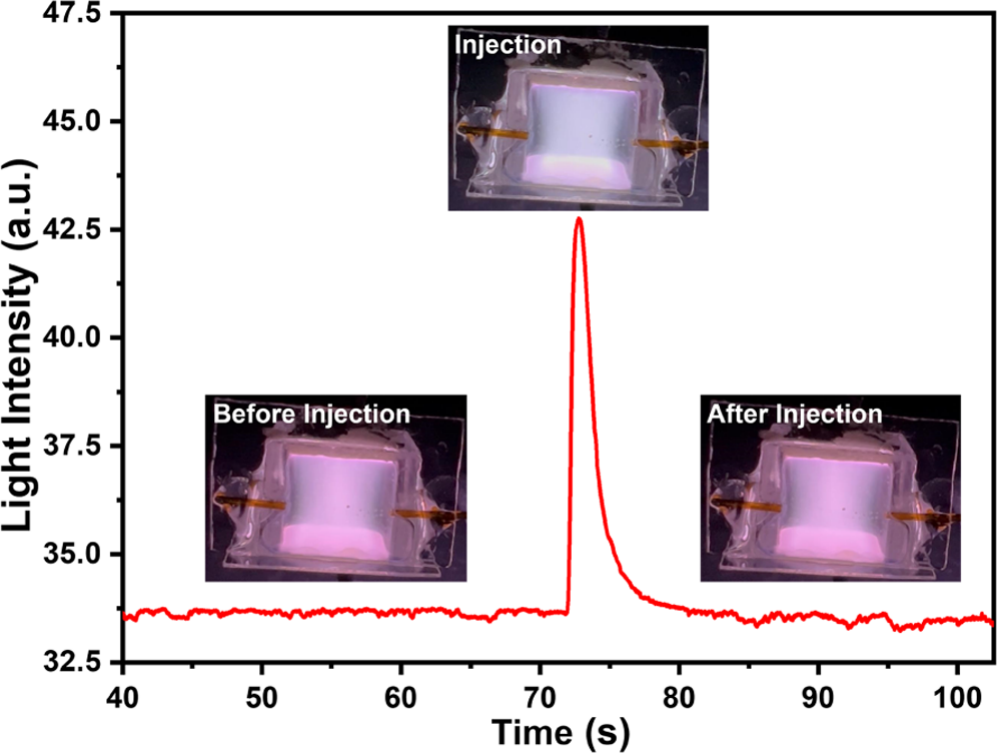
Image light intensity change vs time plot generated from the recorded video after *n*-heptane injection. Device images show the resulting plasma color changes before, during, and after sample injection.

The light intensity and color of the plasma both change when different VOCs are injected into the plasma chamber via a GC column. Figure 5 shows the change in plasma image light intensity calculated using the RGB color space method. The plasma color changes after the injection and elution of 313 ng *n*-heptane sample from the column into the glass chamber. Before *n*-heptane injection, the plasma color is close to lilac and the baseline image light intensity value remains stable. After n-heptane injection, the plasma light intensity increases to a peak value within approx. 1 s and returns to the baseline level after approximately 6 s. This clearly demonstrates that our new method has a very quick response time. Therefore, both plasma color change and the light intensity (calculated from this color change) can be used to demonstrate the presence of VOCs. The image light intensity value change calculated based on two other color space models i.e., HSL (Figure S10a) and HSV (Figure S10b), show the same peak position (elution time of the sample) and a very similar peak height as the RGB model. However, the baseline image light intensity values and fluctuation within the baseline differ. The image light intensity change plot based on HSV color space has the highest baseline height and fluctuation levels. Moreover, the results based on the RGB color space have the lowest fluctuations in baseline values, while the HSL color space model results are in the middle. In addition, the lower baseline fluctuation level makes the RGB image light intensity change easier to observe, especially when the mass of injected VOC is low. Therefore, we only used the RGB color space model to calculate the minimum detection limit of our sensor.

**Figure 6:**
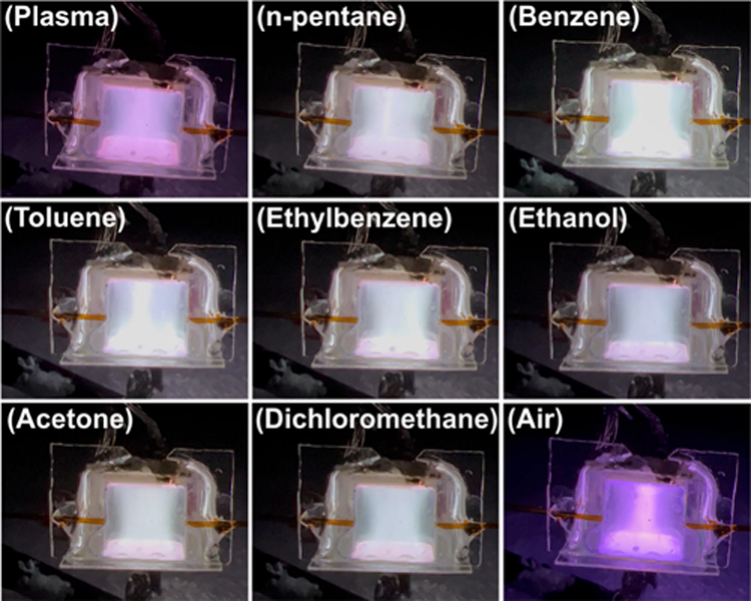
Demonstration of change in color of helium plasma for different sets of VOCs and air sample injection.

Figure 6 depicts snapshots of the μHDBD-PID device after various compounds including alkane (*n*-pentane), aromatics (benzene, toluene, and ethylbenzene), polar (ethanol, acetone, and dichloromethane) compounds, and air were injected into the plasma chamber. These plasma images were extracted from the frame with the highest image light intensity value using a MATLAB program. The *n*-pentane injection mass was approx. 626 ng, resulting in a white plasma color. Similar white color plasma was observed for other alkanes, such as *n*-hexane (655 ng), *n*-heptane (684 ng), *n*-octane (703 ng), and *n*-nonane (718 ng) (Figure S11). After 219 ng benzene was injected, the plasma color changed to white with a light green tint due to the aromatic ring structure.^72^ Both toluene and ethylbenzene, with an injection mass of 217 ng, also resulted in a color change dominated by white. The polar organic compounds also exhibited different colors from the initial helium plasma.

Ethanol (395 ng) caused the plasma color to change from lilac to white with a very light purple color hue, owing to the presence of both ethyl and hydroxyl groups in its molecular structure.^73^ Acetone (392 ng) caused the plasma to become white but with a very light blue color, mainly contributed by carbonyl in its molecular structure.^74^ For dichloromethane, a very light green tint was visible in the white-dominated plasma due to the chlorine atoms being ionized into chloride ions.^75^ Injecting ambient air into the plasma chamber resulted in two major peaks, and the plasma color changed from lilac to dark purple. The appearance of dark purple was due to the ionization of nitrogen in air sample. The IP of nitrogen is 15.58 eV,^76^ verifying the μHDBD-PID’s ability to ionize gases with high IPs and demonstrating the viability of our detection scheme to identify different classes of compounds. We are also conducting further tests to determine if these specific color changes can be used to identify specific sets of compounds, such as benzene, nitrogen, and oxygen.

**Figure 7:**
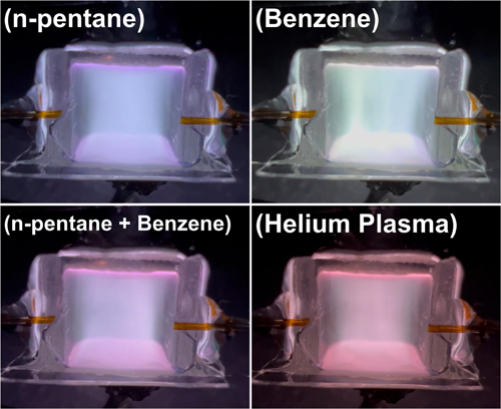
Helium plasma light intensity of *n*-pentane (0.4 μL), benzene (0.4 μL), and their mixture (0.8 μL, volume ratio 1:1). The GC split ratio was set to 200, inlet pressure to 20 psi, and the device was directly connected with a capillary tube.

We also connected a new device directly with a capillary tube to explore color changes in plasma when two compounds are injected together. For this test, we selected n-pentane, benzene, and their mixture (volume ratio 1:1) due to the relatively noticeable difference in color change between *n*-pentane and benzene. The figures corresponding to the image light intensity peaks of each injection are shown in Figure 7. The plasma color generated by the device directly connected via a capillary tube is still close to lilac. As shown in Figure 7, the color changes of *n*-pentane, benzene, and their mixture are different. The plasma color after the injection of *n*-pentane is still white, and the plasma color after the injection of benzene is white with a light green tint. The plasma color after the mixture is injected changes to a light purple. To compare and understand the color changes of these compounds after injection, we also provide the RGB values (Table S1) corresponding to each image in Figure 7, along with 3D plots of this data (Figure S12). Table S1 and Figure S12 show an obvious difference in RGB values between the images of helium plasma (baseline) and *n*-pentane, benzene, and their mixture. The injection of all three substances increased the RGB values. The change in RGB values from the baseline for n-pentane is the smallest, and benzene shows the largest change in RGB values, while the RGB values of their mixtures are between these values (Figure S12).

It is important to note that the illumination distribution within the chamber and color differences in images of different compounds do not determine the image light intensity. Instead, the image light intensity is calculated using eq 1, which is based on the image RGB values. Therefore, there is no direct relationship between the image’s illumination distribution, color, and its light intensity. It is possible for images with different illumination distributions or colors, such as white and white with a light purple tint, to have the same image light intensity values.

### 3.3 Limit of Detection (LoD) and Linearity Range

**Figure 8:**
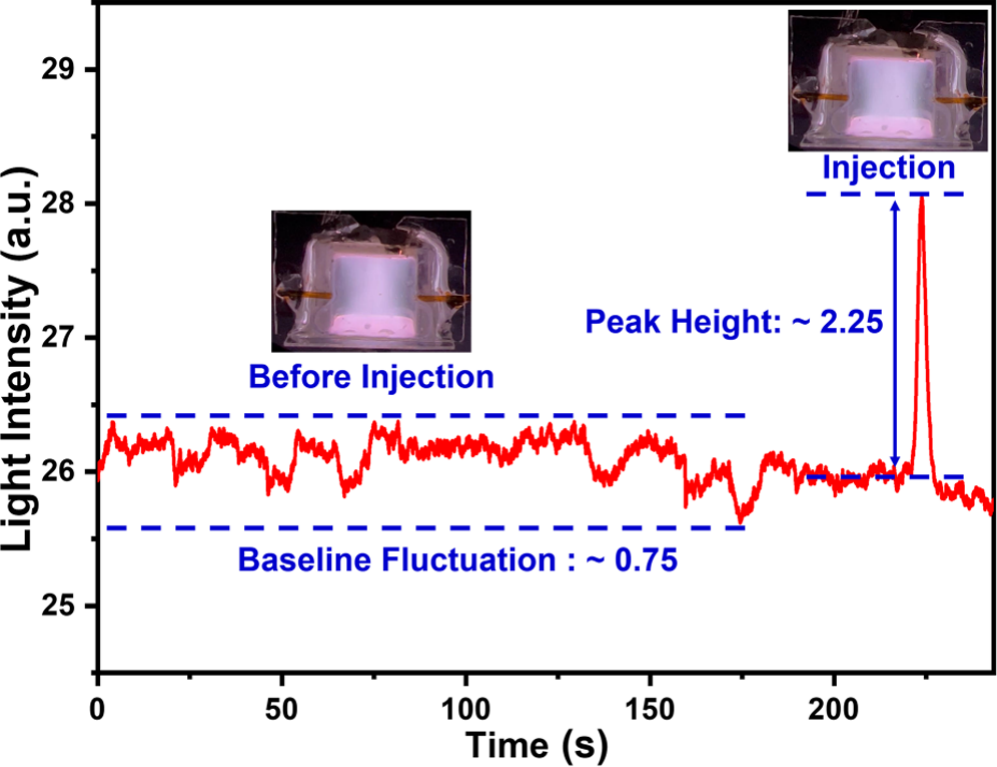
Limit of detection is calculated as three times the baseline signal fluctuation. For example, the limit of detection of 43 ng is calculated for toluene.

The random fluctuations observed in the baseline image light intensity are considered noise and determine the minimum detection limit of our method. We calculated the limit of detection of each compound by multiplying the baseline fluctuation range (maximum minus minimum) with three, as per previously reported work in this area.^10^ Moreover, since the results based on the RGB color space provide the lowest baseline fluctuation compared to both the HSL and HSV color space models, we used RGB-based image light intensity data to measure LoD. Figure 8 shows the image light intensity change calculated based on the RGB color space model when 43 ng of toluene was injected into the plasma chamber via a 30 m long GC column.

Before injection, the μHDBD-PID was operated for 10 min to ensure the plasma remained stable. Toluene was injected through the GC injection port at ∼12 s, and a light intensity peak with a height of ∼2.25 occurred after ∼202 s. The maximum fluctuation of the plasma over the whole process is ∼0.75, so the signal-to-noise ratio is ∼3. It is important to note that the plasma image light intensity baseline height is lower compared to Figure 5 since the LoD result presented here is generated using a repaired device and a slightly different position of the camera.

We fabricated three devices and conducted LoD tests to evaluate device-to-device variations. In our experiments, the position of the camera refers to the distance between the iPhone lens and the observation window of the plasma chamber, which was fixed at approx. 1 cm, as mentioned earlier. Increasing the distance to 5 cm makes it difficult to capture light intensity fluctuations, resulting in a higher LoD.

We measured 11 different chemicals using three devices and calculated the average value as the LoD. The LoD results with their standard deviation (*n* = 3) are presented in Table 2. Among the three classes of VOCs used in this study, alkanes have the highest LoDs, while the aromatic compounds have the lowest LoDs. *N*-pentane within alkanes has the highest LoD among all chemicals, which can be attributed to its ionization potential. Despite having a LoD of 10 ng, our first-generation detector’s sensitivity is at least three orders of magnitude lower than the state-of-the-art μHDBD-PIDs (pg-level) that use electrical signal output.^9,11,20^

**Table 2:** Limit of Detections with Standard Deviation (*n* = 3), Boiling Points, Ionization Potentials, and Linearity Ranges of 11 VOCs.^11,20,76^^a^

compound	average LoD (ng)	standard deviation (*n* = 3)	boiling point (°C)	IP (eV)	linearity range (ng)
*n*-pentane	316	42	36.06	10.35	316–1252
*n*-hexane	142	16	68.72	10.18	142–1310
*n*-heptane	154	14	98.38	10.08	154–1368
*n*-octane	164	17	125.62	9.82	164–1406
*n*-nonane	174	8	150.8	9.71	174–1436
benzene	10	1	80.08	9.25	10–88
toluene	46	4	110.6	8.82	46–217
ethylbenzene	79	10	136.2	8.77	79–217
ethanol	18	2	78.24	10.43	18–789
acetone	26	9	56.08	9.69	26–784
dichloromethane	144	16	39.8	11.35	144–1330

a The LoD values reported in the table are the average measured from three devices.

We also performed linearity tests on 11 VOCs (Table 2). For our device, when the VOCs injection volume is higher than a certain value, the device will saturate with no more increase in plasma light intensity (excessive injection volume can also cause a decrease in plasma light intensity). The range of linearity is based on RGB color space image light intensity. However, we have observed that the image light intensity of HSL and HSV color spaces is consistent with RGB data. Table 2 shows that our device has a relatively broad linearity range for alkanes and a relatively narrow linearity range for aromatic compounds.

Our detection scheme provides an exciting opportunity to identify different sets of compounds based on the color change of plasma. As a new μHDBD-PID that relies on image light intensity change as a readout method, there are several factors limiting the LoD of devices. First, we are only using a visible light camera (iPhone 13 Pro, spectrum range: 380–700 nm) for our experiments. Additionally, the glass observation window, made by Schott D263M Glass, has a spectral transmittance of 91.7% in the wavelength range of 250–3000 nm.^77^ Due to these limitations, the light wavelength range that our detection scheme can capture is limited to 380–700 nm. Moreover, the micro-focusing capability of our existing smartphone camera also limits the collection efficiency of light. Similarly, the electromagnetic environment generated by plasma and high-voltage electrodes also limits the minimum distance between the camera lens and the viewing window. These shortcomings also provide new research opportunities to further improve the LoD by experimenting with both device packaging and camera type.

To further enhance the sensitivity of the readout signal, several optimizations can be implemented. The first option is to replace the iPhone 13 Pro with a well-packaged camera that can capture photons with a wide range of wavelengths, from infrared to UV. Additionally, the Schott D263M Glass can be substituted with a glass having a wider transmittance range, such as UV Fused Silica (UVFS, 195–2100 nm) or CaF_2_ (170–8000 nm)^78^ These glasses possess excellent chemical resistance and are extensively used in advanced optical systems.^79,80^ More significantly, both UV Fused Silica and CaF_2_ can resist plasma etching to a certain extent.^81,82^ To confirm and compare the performance of these potential glass materials, further testing is required.

### 3.4 Performance Evaluation with a GC Column

**Figure 9:**
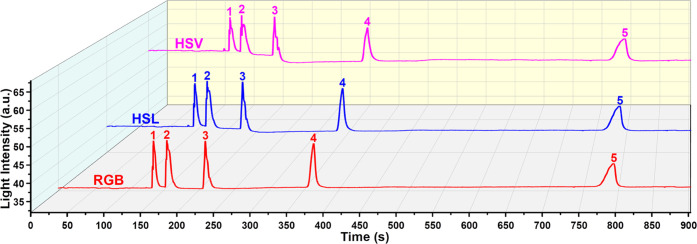
μHDBD-PID detection response of alkane mixture (equal volume mixture of five *n*-alkanes). The injection volume is 0.6 μL with a split ratio of 200. Elution order: (1) *n*-pentane; (2) *n*-hexane; (3) *n*-heptane; (4) *n*-octane; (5) *n*-nonane.

**Figure 10:**
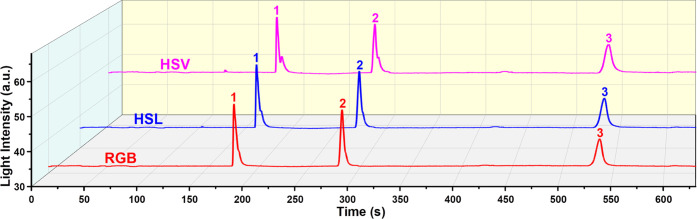
μHDBD-PID detection response for a mixture of aromatic compounds (equal volume mixture of benzene, toluene, and ethylbenzene). The sample injection volume is 0.6 μL with a split ratio of 100. Elution order: (1) benzene; (2) toluene; (3) ethylbenzene.

**Figure 11:**
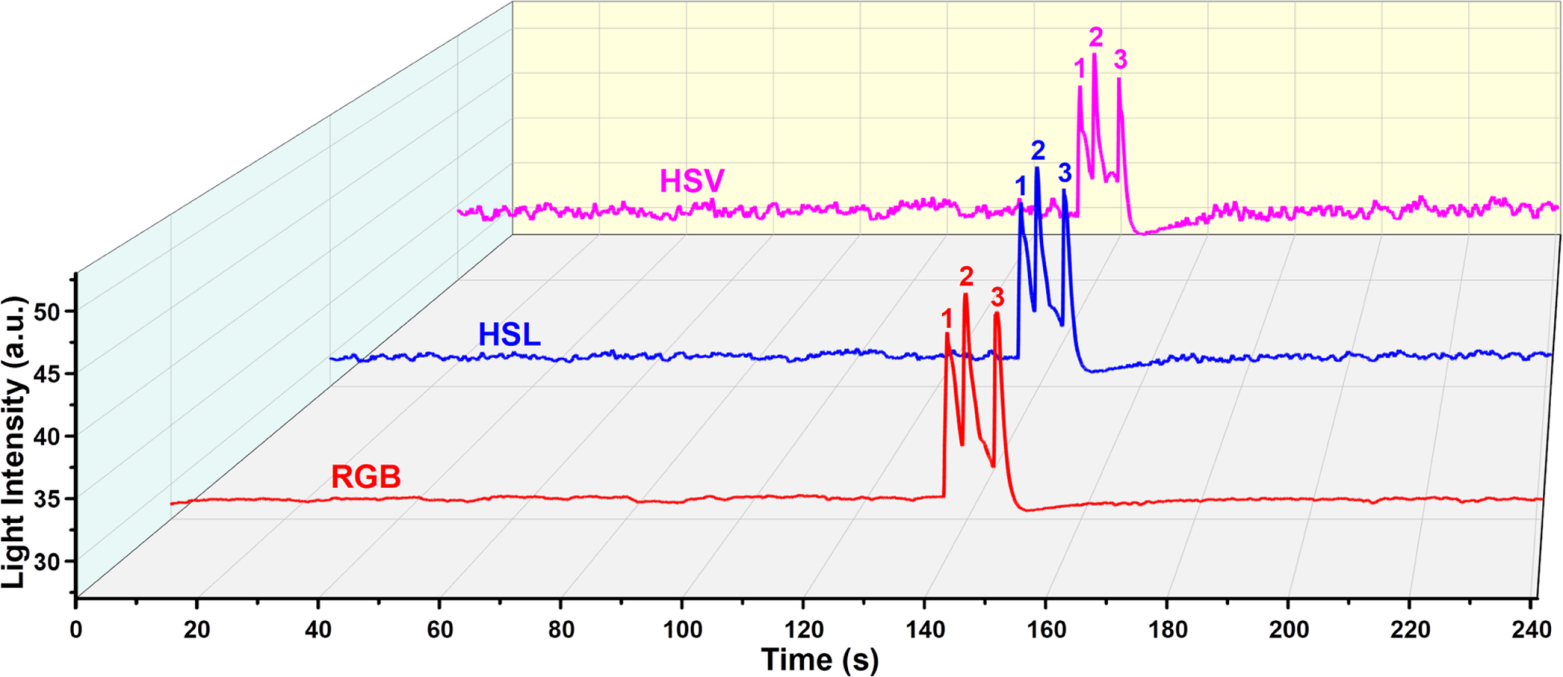
μHDBD-PID response for a mixture of polar compounds (equal volume mixture of ethanol, acetone, and dichloromethane). The injection volume is 0.3 μL with a split ratio of 200. Elution order: (1) ethanol; (2) acetone; and (3) dichloromethane.

The chromatographic performance of μHDBD-PID was demonstrated by testing three separate mixtures of alkane, aromatics, and polar compounds using a GC column. The injection port pressure was set to 20 Psi, and the inlet temperature of the GC system was set to 270 °C. All three mixtures are prepared by mixing equal volumes of liquids. Figures 9–11 show the detector response to mixtures of alkane, aromatic, and polar compounds. In Figure 9, five image light intensity peaks can be clearly seen based on the three-color space models. The detector also demonstrated excellent detection capability for aromatic ring compounds and polar organic molecules, as shown in Figures 10 and 11. Compared to the HSL and HSV color spaces, the RGB color space has a better peak shape (better symmetry) with less peak splitting (Figures 9–11). The difference among the three-color space models based on baseline fluctuation level has been previously discussed in Figures 5 and S10. For the detection results of the mixtures, we observed the same difference. Briefly, the HSL and HSV color spaces have larger fluctuations than RGB. The difference in baseline fluctuation level caused by different color space calculation methods is more obvious in the detection results of polar organics (Figure 11). From the RGB to the HSV method, the increase in baseline fluctuations is more obvious. Although there are some differences in the image light intensity plots of these three-color spaces, these results demonstrate that all three models can be used for the detection of VOCs. To explore the potential of our detection scheme for identifying compounds, we analyzed the RGB values of all 11 VOCs corresponding to their peaks. Tables S2–S4 show the RGB values of 11 VOCs corresponding to their image peaks (Figures 9–11) and the baseline RGB values. In addition, a 3D plot (Figure S13) was created to visualize the differences in RGB values. The distribution of alkanes, aromatic, and polar organic compounds in RGB 3D space is indeed different, as shown in Figure S13. However, there are exceptions, such as the positions of *n*-nonane and ethylbenzene, which are not close to their class of compounds. While these results demonstrate the potential of using RGB values to differentiate different sets of VOCs, identifying VOCs using RGB values or any other color representation system requires generating a comprehensive database for each compound and a high-fidelity image processing algorithm to obtain reliable results.

Our first-generation device suffers from the problem of peak splitting, as can be seen in Figure 9 (*n*-pentane, *n*-hexane, and *n*-heptane), Figure 10 (benzene and toluene), and Figure 11 (ethanol and acetone). This is not an artifact generated during image post-processing nor caused by degradation due to the etching of the viewing glass. As shown in Figure 9, after the appearance of peak splitting in the peaks of *n*-pentane, *n*-hexane, and *n*-heptane, the peaks of *n*-octane and *n*-nonane remain relatively symmetric. We hypothesize that peak splitting is caused by the nonuniform flow of VOCs and helium inside the plasma chamber. The internal design of our chamber is not optimized to align with gas flow dynamics, which means that VOCs cannot diffuse uniformly throughout the internal space instantly and be ionized within a short period of time. When VOCs enter the ionization chamber, the molecules are immediately diluted by helium, and the VOCs’ ionization rate by helium plasma is not uniform. VOCs flowing into regions with a high electron density, such as the central region of helium plasma, will be rapidly ionized and emit a large number of photons. In contrast, VOCs flowing into low electron density regions will emit relatively few photons. Therefore, the photons emitted by VOCs are not uniform, causing the chromatographic peaks to appear split. However, due to the small size of our chamber, the spatial distribution of VOCs inside the chamber is relatively small, so the ionization luminescence of VOCs can still maintain some degree of uniformity. In the future, we plan to optimize the chamber design and eliminate peak splitting.

### 3.5 Device Reliability and Long-Term Operation

The high reactivity of the helium plasma caused the glue between the glass viewing window and the plasma chamber interface to be etched/removed during testing. The whole process of etching is shown in Figure S14. From 0 to 3 h, there is no noticeable change at the interface. After 5 h of μHDBD-PID operation, small etching spots started to appear at the interface and slowly developed into etched channels after 9 h. After 10 h of operation and multiple sample injections, these etched channels became visible and broke through the interface. This did not damage the device but left a small black area. This damage was easily fixed by removing the burnt areas with a small needle and resealing the damaged interface with UV-curable glue. We are currently working on a more resilient solution by permanently bonding the glass plate and plasma chamber together at a high temperature.

**Figure 12:**
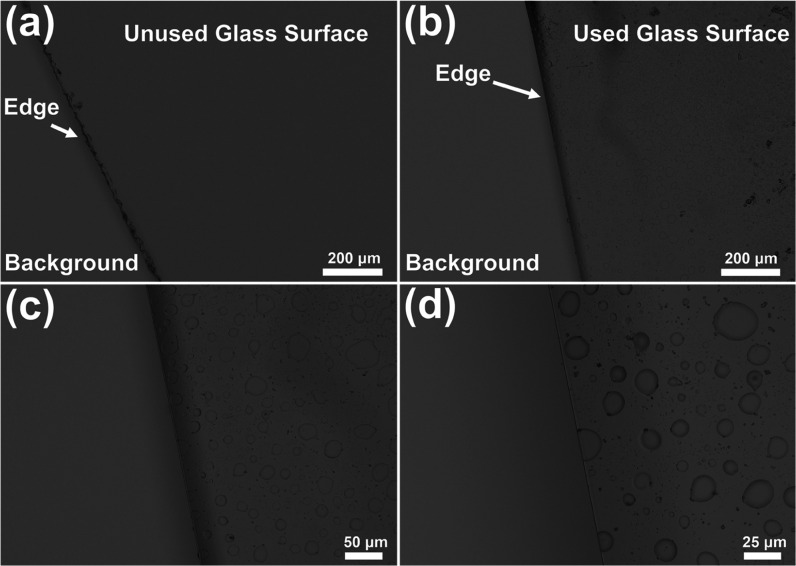
Optical images of a viewing glass window attached to the plasma chamber. (a) Image of a new unused glass plate surface (magnification 200×). (b–d) Optical images of glass plate surface at different magnification levels after 20 h of plasma operation.

To observe the damage caused to the viewing glass window by constant exposure to plasma, we compared a new, unused glass plate with the used one. We disassembled a viewing glass window from one of the working devices, and Figure 12a shows the optical image of the unused glass plate surface. The surface of this new glass plate is very smooth, without any visible defects. In contrast, many circular pit defects and small particles are observed on the surface of the used glass plate (Figure 12b–d) after 20 h of operation. These ring-shaped pits are caused by the constant impact and etching of plasma, and the surface particles seen in the image are residue from different chemicals injected into the plasma chamber. This shows that the transparency of the viewing window of the μHDBD-PID will eventually decrease due to plasma etching and accumulation of chemicals on the surface. Low transparency will decrease the light intensity signal received by the camera and affect detector performance. Therefore, it is necessary to replace the observation window regularly for our first-generation devices.

For our current device, the replacement procedure is simple, and the cost of a new glass plate is very low (a single glass slide can make six viewing windows), which means the detector’s operation life can be continuously extended. In the future, we are planning to select a more chemically resistant glass substrate as a viewing window.

## 4 Conclusions

This paper presents a new image/video analysis-based μHDBD-PID with new detector signal collection and manufacturing methods that differ from other μHDBD-PIDs. Our new technique validates the possibility of using a change in plasma color, which is converted to an equivalent image light intensity signal, as a signal output for the detector. The μHDBD-PID demonstrates excellent detection performance for mixtures of alkanes, aromatics, and polar organics, indicating its potential for GC applications. In the future, we will focus on improving the detector lifetime and LoD. To enhance the long-term stability of our detector, we can use an etch-resistant glass and permanently bond the viewing window with the plasma chamber. Similarly, replacing the visible light camera with a high-resolution portable multispectral camera and reducing the distance between the camera and the device can further improve detector sensitivity.

## Supporting Information Available

3D CAD model used for printing plasma chamber with detailed dimensions (Figures S1 and S2); photo of μHDBD-PID assembly (Figure S3); power supply circuit diagram (Figure S4a) and image (Figure S4b) and thermal images of different power supply components (Figure S4c); detailed flowchart of Python code used for processing recorded video (Figure S5); COMSOL 3D helium flow simulation model (Figure S6) with velocity field and simulation results (Figure S7); schematic diagram of COMSOL 1D and 2D model area selection, built models (Figure S8) and simulation results (Figure S9); image light intensity change plots generated using HSL and HSV color spaces when *n*-heptane injected into plasma chamber (Figure S10); plasma images corresponding to image light intensity peak values when *n*-hexane, *n*-heptane, *n*-octane, and *n*-nonane were injected into chamber (Figure S11); RGB values of image light intensity peaks corresponding to *n*-pentane, benzene, *n*-pentane, and benzene mixture (Table S1) and 3D plot of RGB values (Figure S12); RGB values of baseline plasma image and pictures with image light intensity peak corresponding 11 VOCs (Table S2–S4) and 3D plot of RGB values (Figure S13); device change operation from 0 to 10 h and the damage observed at interface of chamber and viewing window (Figure S14); and Python code for video processing and MATLAB code (PDF)
