# Supplementary material for: Colorimetric Signal Readout for the Detection of Volatile Organic Compounds Using a Printable Glass-Based Dielectric Barrier Discharge-Type Helium Plasma Detector
Source: ACS Meas Sci Au. 2023 May 30;3(4):287–300. doi: 10.1021/acsmeasuresciau.3c00012 (PMC10436375; doi:10.1021/acsmeasuresciau.3c00012)
Supplement: Supplementary file 1 — tg3c00012_si_001.pdf [file tg3c00012_si_001.pdf]

Supporting Information for Colorimetric Signal Readout for the Detection of Volatile OrganicCompounds Using a Printable Glass-Based Dielectric Barrier Discharge-Type Helium Plasma Detector

Jingqin Mao[†], Longze Liu[†], Yahya, Atwa[†], Junming Hou[§], Zhenxun Wu[ʃ], Hamza Shakeel[†*].

† School of Electronics, Electrical Engineering and Computer Science, Queen's University Belfast, Belfast BT7 1NN, United Kingdom

§ State Key Laboratory of Millimeter Waves, School of Information Science and Engineering, Southeast University, Nanjing 210096, China

ʃ Queen's Management School, Queen's University Belfast, Belfast BT7 1NN, United Kingdom

* Corresponding Author, Email address: H.Shakeel@qub.ac.uk

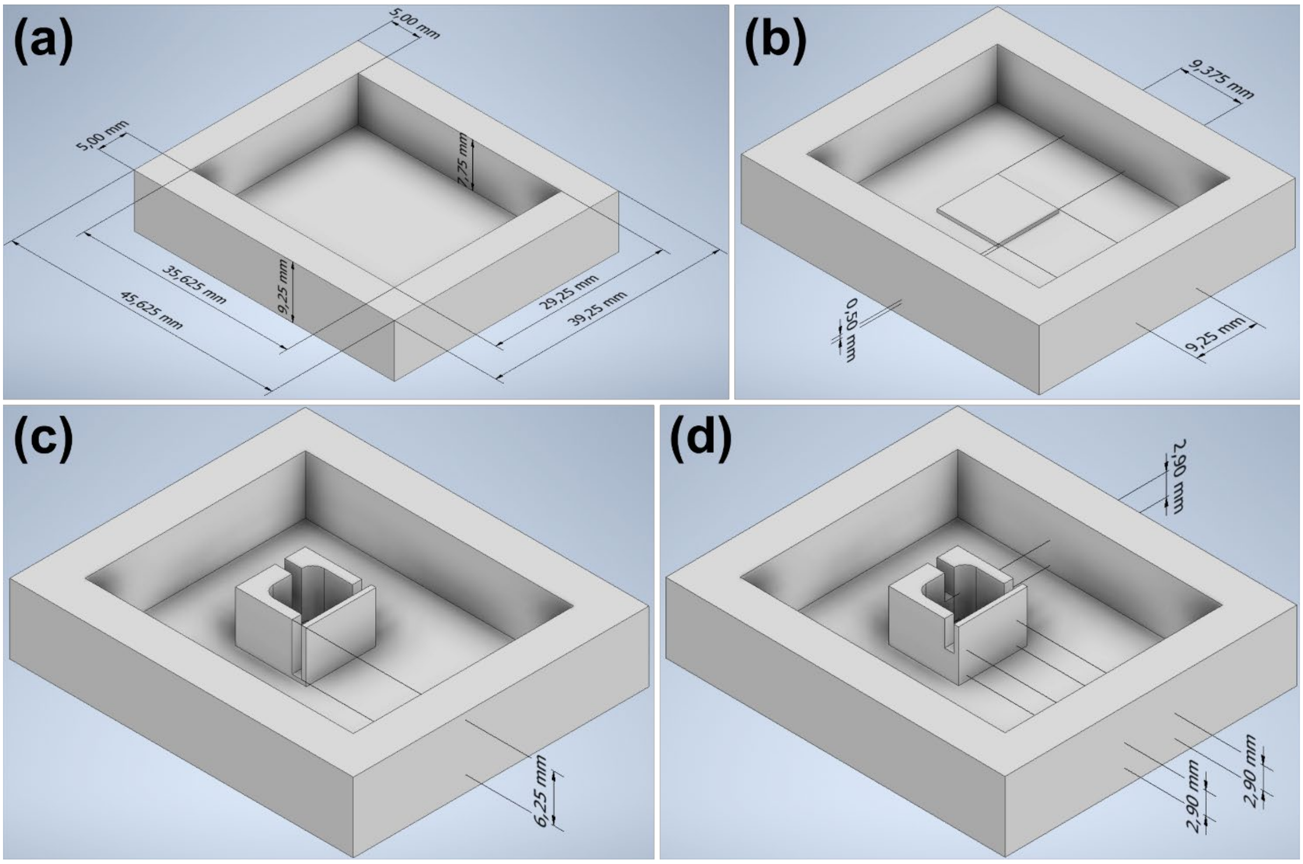

Figure S1. Plasma chamber model design used for 3D printing. (a) A large cavity is first designed which is used to place the actual device model (bottom thickness is 1.5 mm). (b) Dimensions of plasma chamber bottom dielectric plate (thickness: 0.5 mm) inside the cavity. (c-d) critical dimensions of the plasma chamber with inlet, outlets and a dummy front surface which is required to ensure that the structure does not deform during processing.

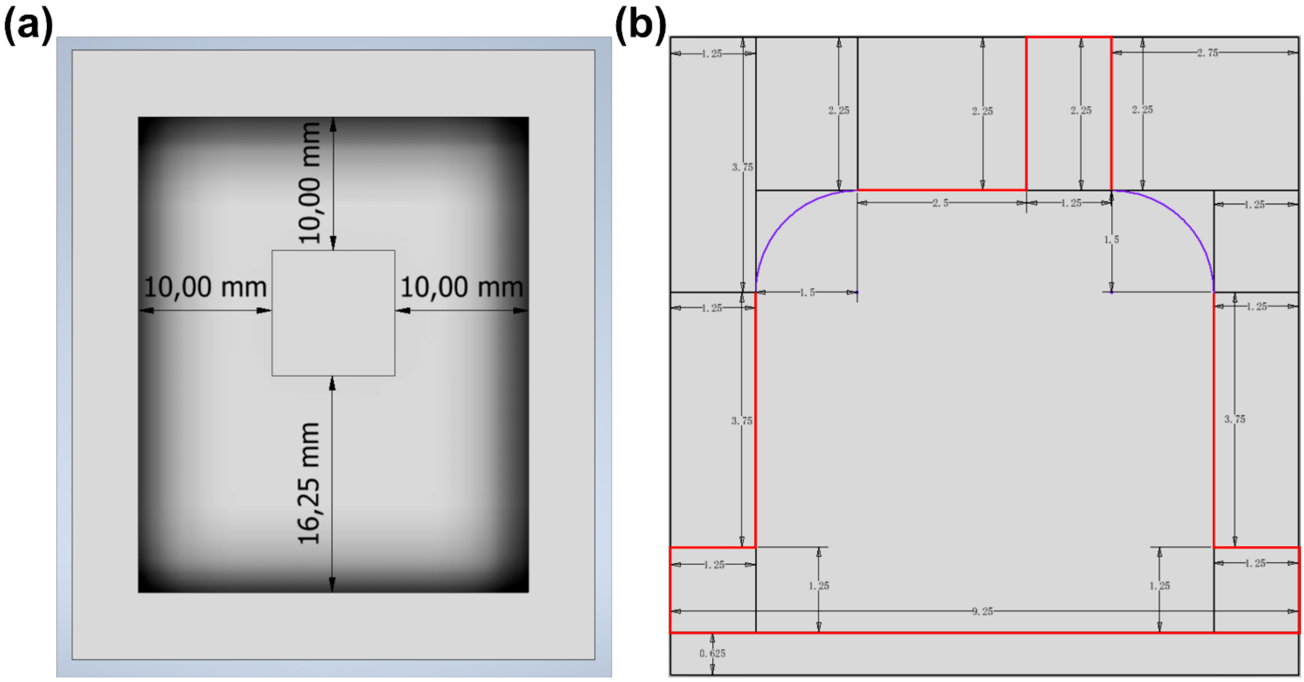

Figure S2. (a) Placement position of the bottom dielectric plate inside the cavity. (b) Detailed dimensions of the bottom dielectric plate. Red lines and two purple quarter circles show the 2D projection of the plasma chamber on the horizontal plane.

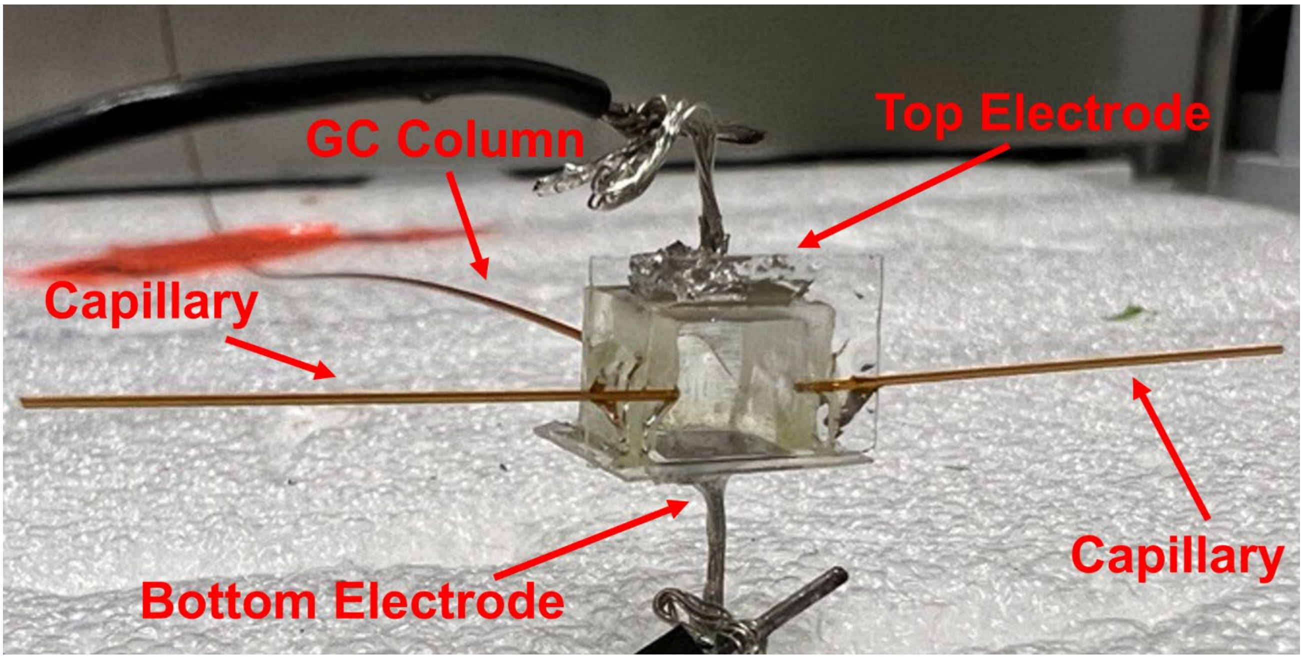

Figure S3. Image of fully assembled plasma chamber connected to a GC column and capillary tubes (outlets).

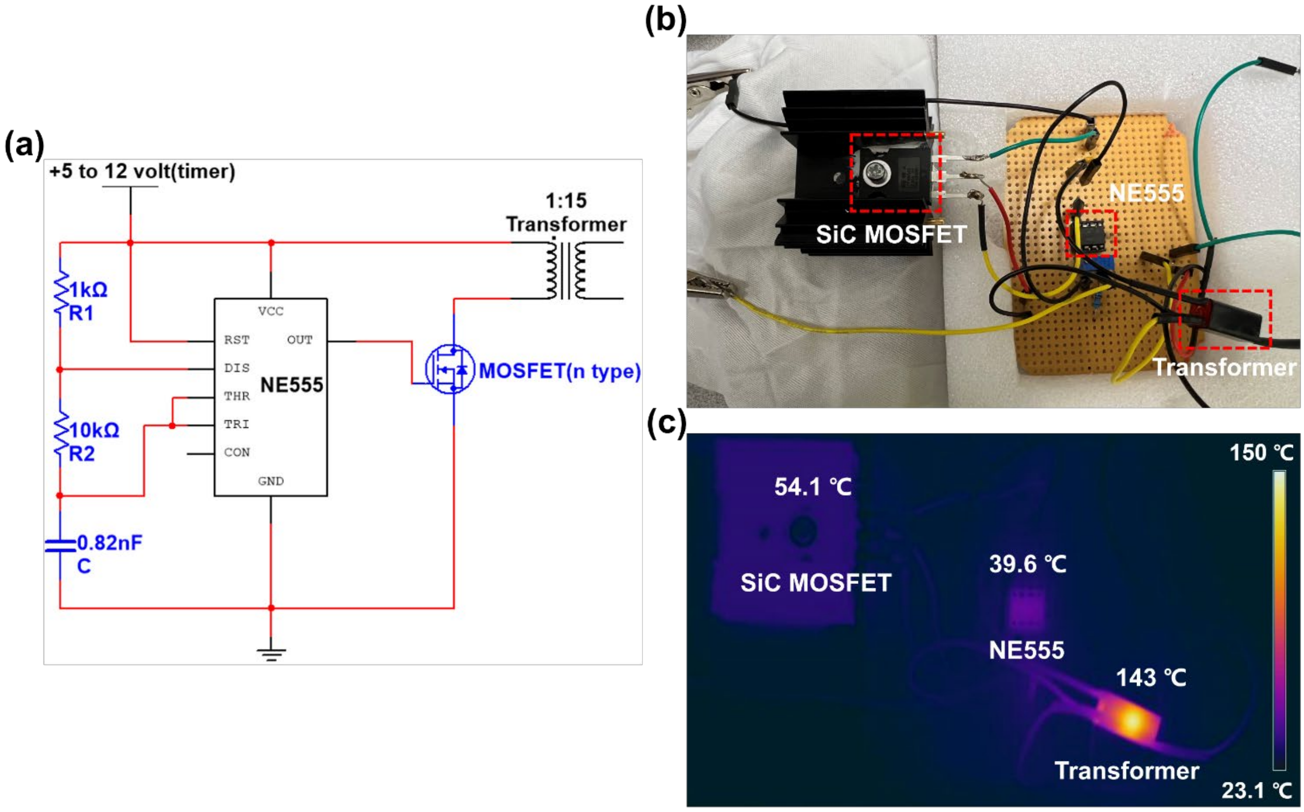

Figure S4. (a) Circuit diagram of high voltage power supply used for plasma generation.[1] (b) Image of fully assembled power supply device unit. (c) Thermal image of power supply device and the working temperatures of SiC MOSFET, NE555 astable multivibrator and ZS1052(H) transformer during operation.

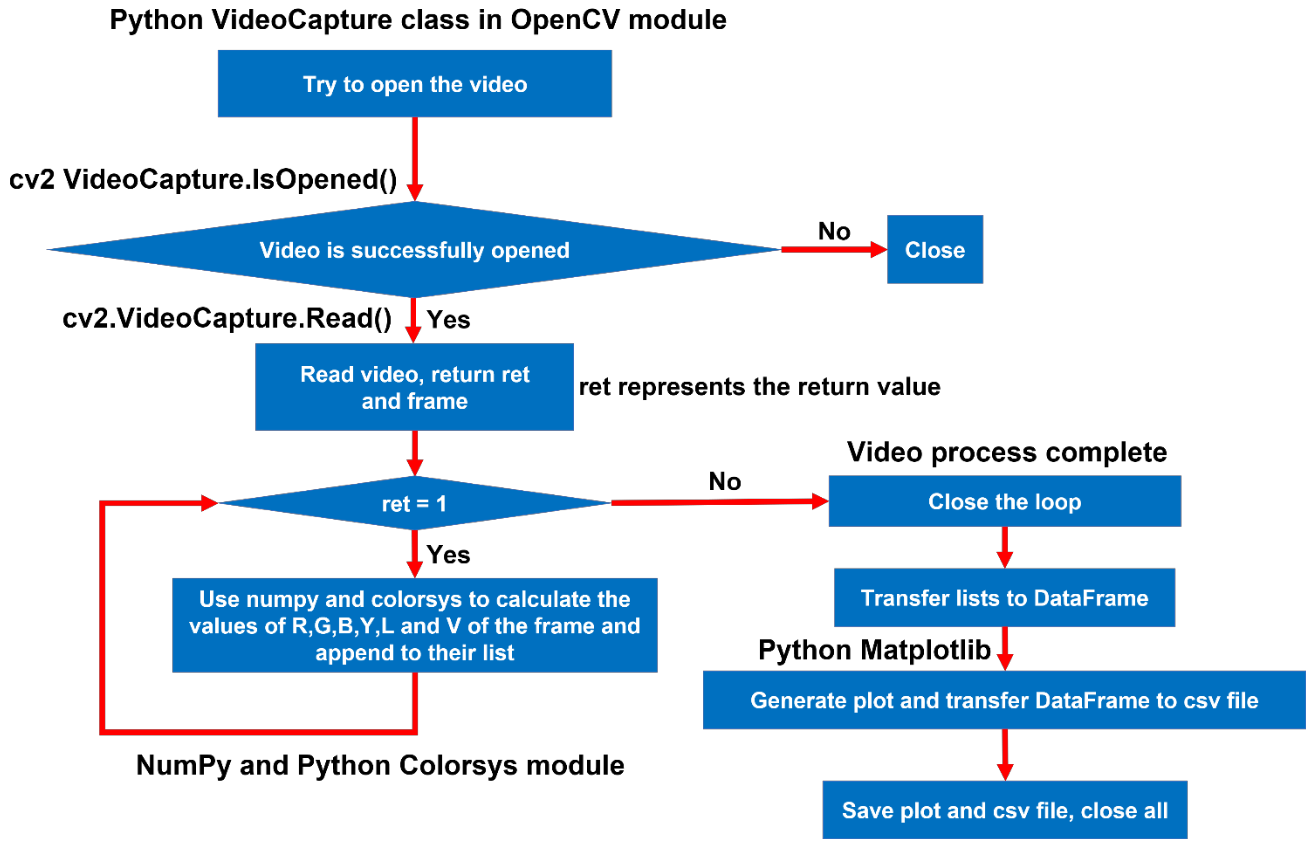

Figure S5. Flow chart showing details of Python code used for processing recorded video.

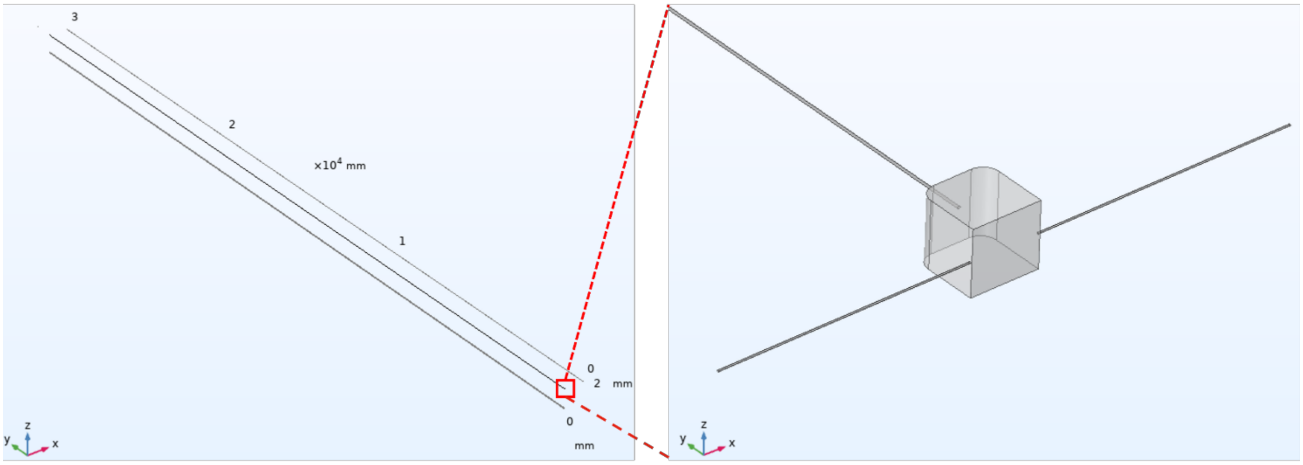

Figure S6. 3D COMSOL simulation model of helium flow through the actual sized plasma chamber.

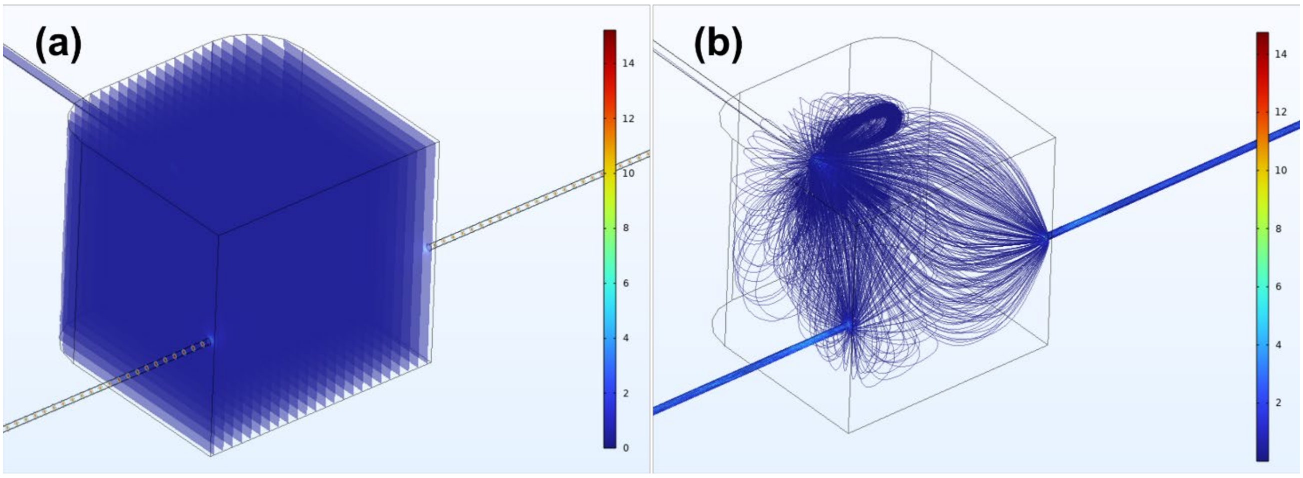

Figure S7. COMSOL 3D helium flow simulations of (a) velocity field and (b) helium flow streamline.

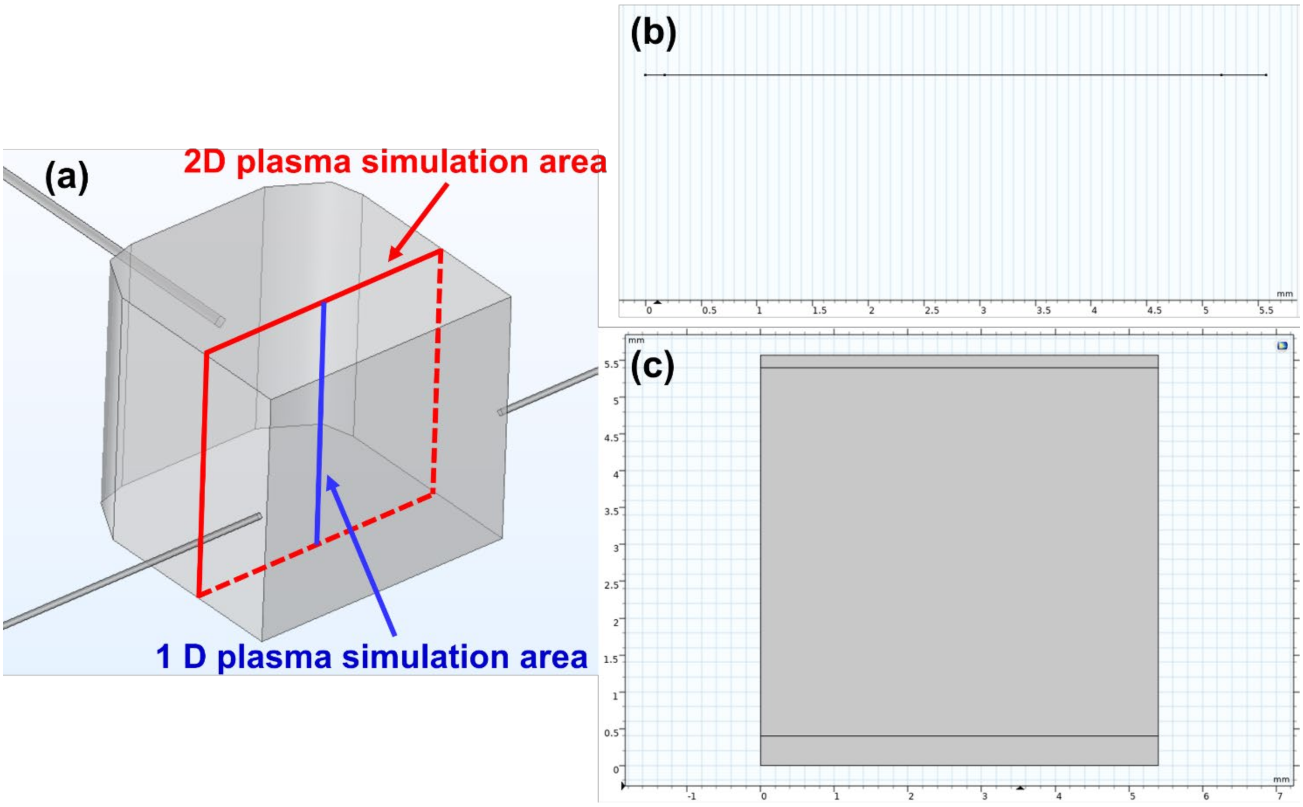

Figure S8. (a) Image shows selected areas from the 3D model used for 1D and 2D COMSOL helium plasma simulations. Geometries of (b) 1D and (c) 2D helium plasma simulation models.

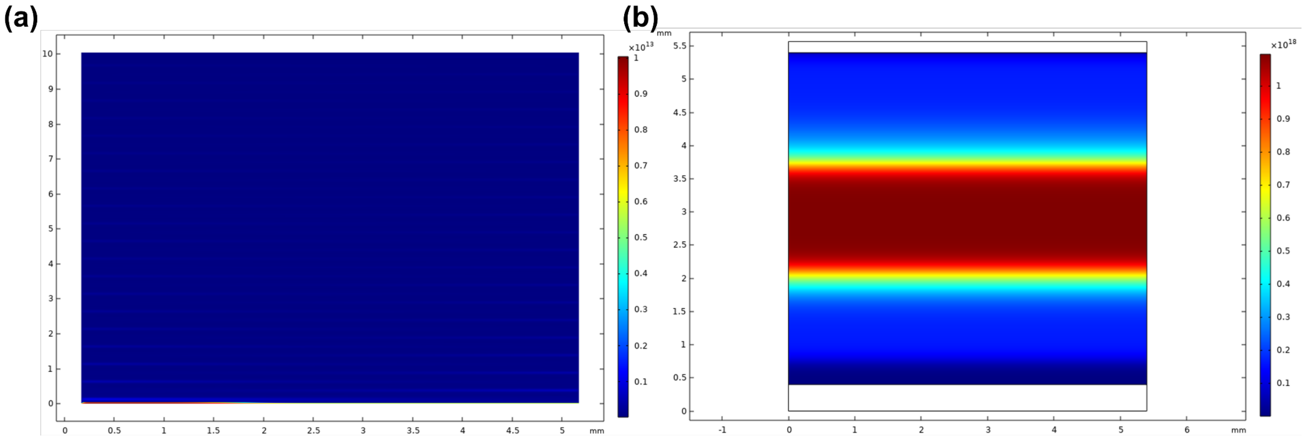

Figure S9. (a) Electron density distribution from 0 to 10th discharge cycles of 1D helium plasma (2D results were generated by 1D parametric extrusion). (b) The electron density distribution of 2D plasma simulation at the 10th discharge cycle when voltage amplitude is 9 kV.

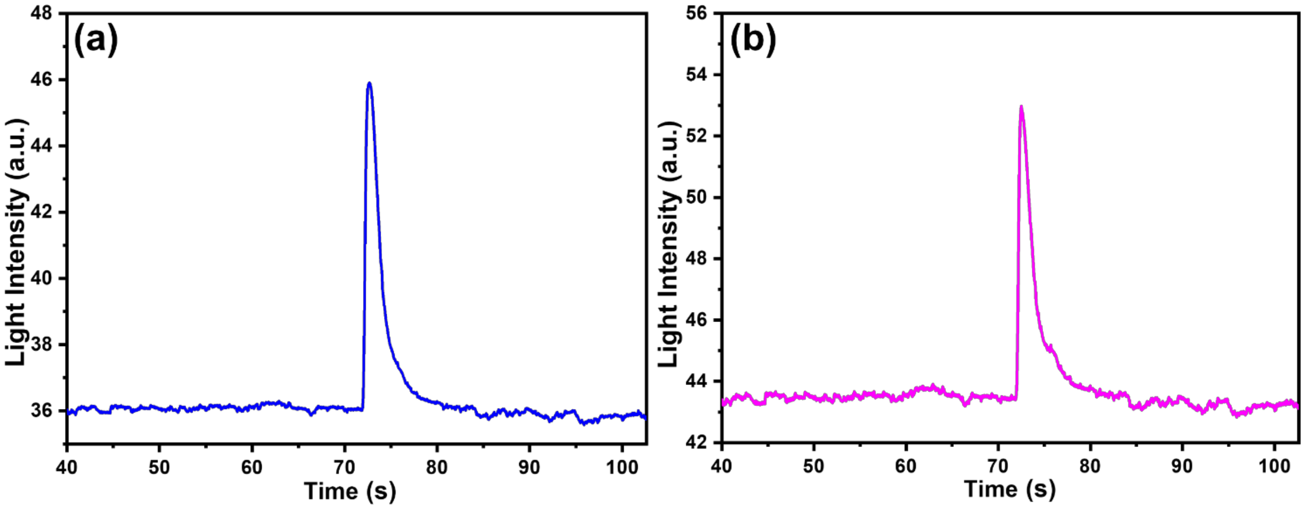

Figure S10. Image light intensity change plots when n-heptane was injected into the plasma chamber. Results based on the (a) HSL and (b) HSV color space models.

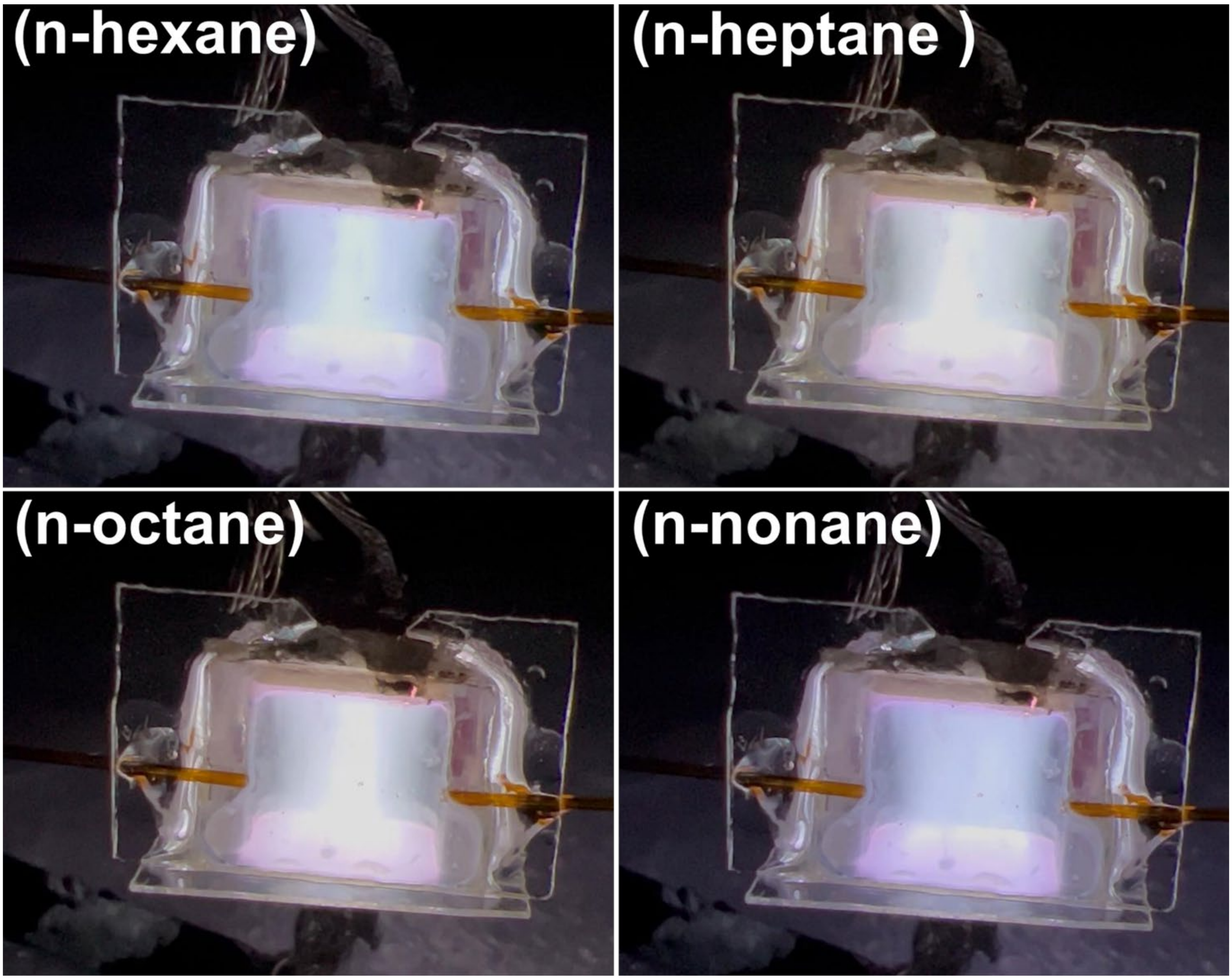

Figure S11. Plasma images corresponding to elution of n-hexane, n-heptane, n-octane, and n-nonane.

Table S1. RGB values of images corresponding to plasma baseline, n-pentane, benzene, and mixture of n-pentane and benzene (Figure 7).

Color	n-pentane	Benzene	Mixture	Baseline
R	35.41	51.59	38.17	30.55
G	32.18	48.53	33.07	23.34
B	44.22	57.68	45.38	27.98

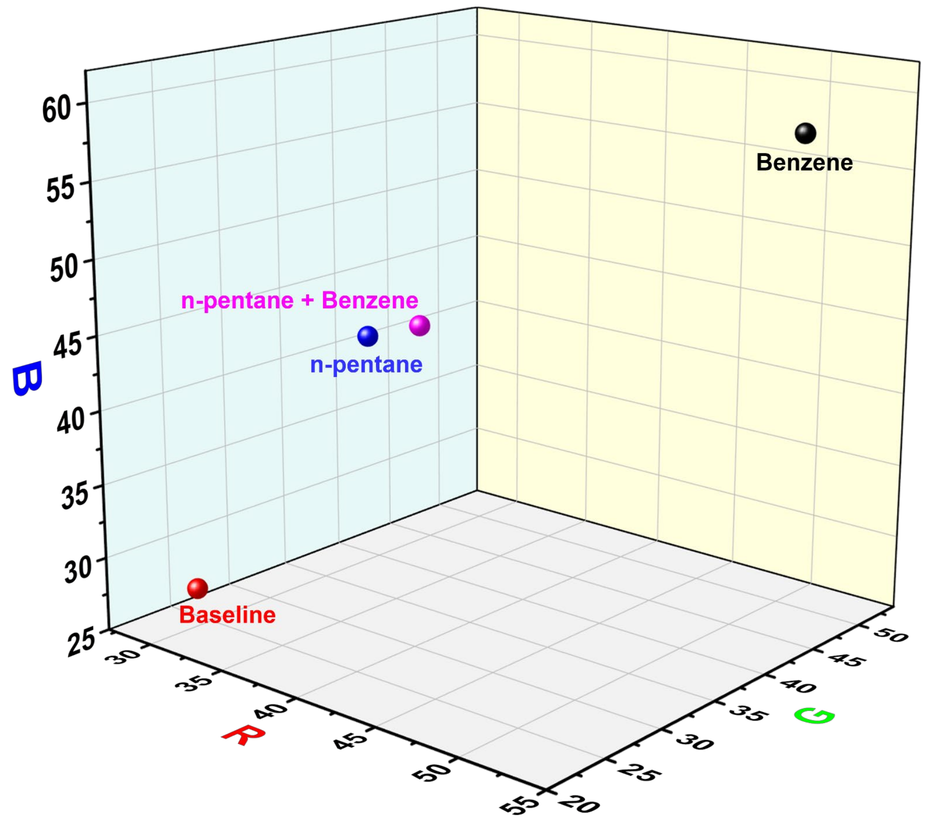

Figure S12. 3D plot of RGB values corresponding to Table S1.

Table S2. RGB values of images corresponding to plasma baseline, n-pentane, n-hexane, n-heptane, n-octane, and n-nonane (Figure 9).

Color	Baseline	n-pentane	n-hexane	n-heptane	n-octane	n-nonane
R	39.37	47.64	47.89	47.99	47.83	44.30
G	25.22	42.14	42.07	41.51	41.10	33.83
B	55.00	64.42	65.60	66.23	62.61	58.97

Table S3. RGB values of images corresponding to plasma baseline, benzene, toluene and ethylbenzene (Figure 10).

Color	Baseline	Benzene	Toluene	Ethylbenzene
R	39.11	52.07	50.82	44.61
G	25.50	46.50	44.76	34.60
B	47.13	65.08	62.77	56.22

Table S4. RGB values of images corresponding to plasma baseline, ethanol, acetone and dichloromethane (Figure 11).

Color	Baseline	Ethanol	Acetone	Dichloromethane
R	33.02	43.39	46.20	45.07
G	25.28	41.87	45.56	44.35
B	34.04	47.39	50.19	45.34

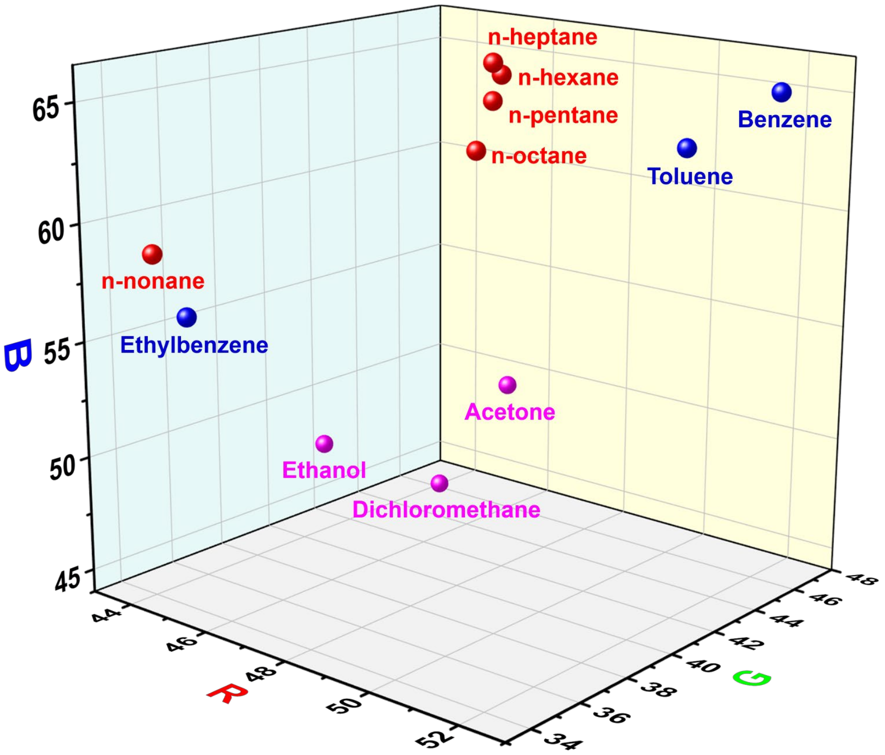

Figure S13. 3D plot of RGB values corresponding to Table S2, Table S3 and Table S4.

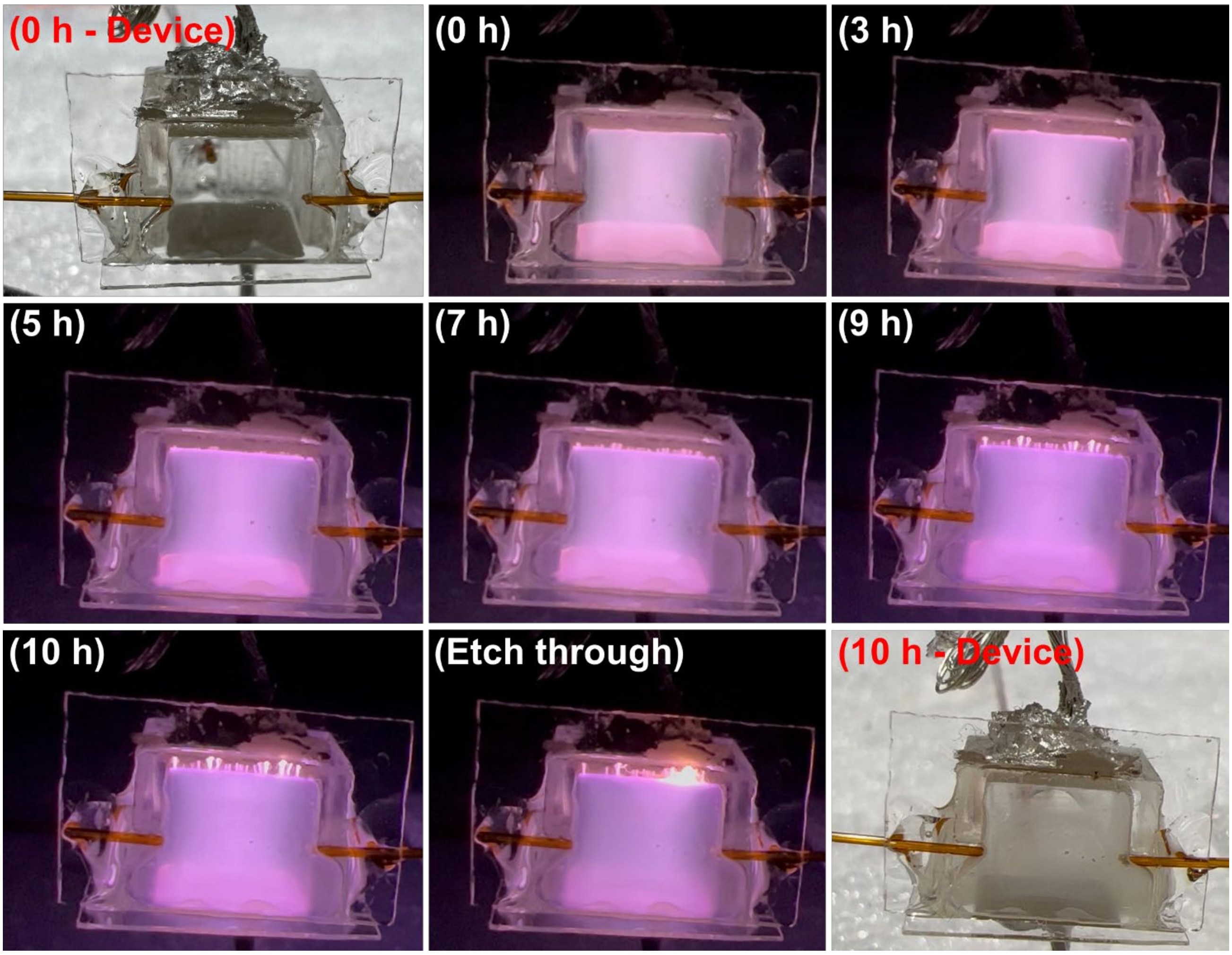

Figure S14. Device operation from 0 to 10 hours and damage observed at the interface of chamber and viewing window.

Python Code for Video Processing

```
import os
import cv2
import colorsys
import pandas as pd
import matplotlib.pyplot as plt

videos_src_path = 'MOV'

videos = os.listdir(videos_src_path)


def csv_and_plot():
    for each_video in videos:
        each_video_full_path = os.path.join(videos_src_path, each_video)
        cap = cv2.VideoCapture(each_video_full_path)

        if cap.isOpened():
            success = True
        else:
            success = False
            print("Read failed!")

        frame_index = 0

        R = []
        G = []
        B = []
        Y = []
        L = []
        V = []

        while success:
            success, frame = cap.read()
            if success:
                B.append(frame[:, :, 0].mean())
                G.append(frame[:, :, 1].mean())
                R.append(frame[:, :, 2].mean())
                Y.append(0.299 * frame[:, :, 2].mean() +
                         0.587 * frame[:, :, 1].mean() +
                         0.114 * frame[:, :, 0].mean())
                L.append(colorsys.rgb_to_hls(frame[:, :, 2].mean()/255,
                                             frame[:, :, 1].mean()/255,
                                             frame[:, :, 0].mean()/255)[1]*255)
                V.append(colorsys.rgb_to_hsv(frame[:, :, 2].mean() / 255,
```

```
                                  frame[:, :, 1].mean() / 255,
                                  frame[:, :, 0].mean() / 255)[2]*255)
        frame_index += 1

    df = pd.DataFrame({'R': R, 'G': G, 'B': B, 'Y': Y, 'L': L, 'V': V})
    plt.plot(df.Y, label='Y')
    plt.title(f"{each_video[:-4]} brightness plot")
    plt.xlabel("img_num")
    plt.ylabel("brightness_value")
    plt.plot(df.L, label='L')
    plt.plot(df.V, label='V')
    plt.legend()
    plt.savefig(f"JPG/plots/{each_video[:-4]} brightness plot.jpg")
    plt.close()
    try:
        df.to_csv(f"CSV/{each_video[:-4]}.csv")
    except:
        raise PermissionError("Close the opened csv first!")


if __name__ == "__main__":
    csv_and_plot()
```

MATLAB Code

```
V = 'L.MOV';
v= VideoReader(V)
numberFrames = get(v,'NumFrames')
%%

delete images/*
%%

starFrame = 1;
endFrame = numberFrames;
for i = 1 : endFrame
   frame = read(v,i);
   imshow(frame);
   outputFile = ['images\image_',num2str(i),'.jpg']
   imwrite(frame,outputFile);
end

%%

file_path =  '.\data\images';%
img_path_list = dir(strcat(file_path,'*.jpg'));
img_num = length(img_path_list);
%%

if img_num > 0
      for j = 1:img_num
         image_name = img_path_list(j).name;
         image =  imread(strcat(file_path,image_name));



      end
end

%%%%%%%%%%%%%%%%%%%%%%%%%%%%%%%%%%%%%%%%%%%%%%%%%%%%%%%%%%%
```

References

(1) Li, M. W.-H.; Ghosh, A.; Sharma, R.; Zhu, H.; Fan, X. Integrated microfluidic helium discharge photoionization detectors. *Sens. Actuators, B* **2021,** *332*, 129504.
